# A nonlocal West Nile virus model with nonlocal free boundary conditions driven by both mosquitoes and birds

**DOI:** 10.1007/s00285-026-02355-9

**Published:** 2026-03-09

**Authors:** Xin Long, Yijun Lou, Wenjie Ni, Taishan Yi

**Affiliations:** 1 School of Mathematics and Statistics, Changsha University of Science and Technology, Changsha, 410114 Hunan China; 2 School of Mathematics (Zhuhai), Sun Yat-Sen University, Zhuhai, 519082 Guangdong China; 3 Department of Applied Mathematics, Hong Kong Polytechnic University, Hong Kong, SAR China; 4 School of Science and Technology, University of New England, Armidale, 2351 NSW Australia

**Keywords:** Nonlocal diffusion, Free boundary, West Nile virus, Spreading, Vanishing, 35R09, 35R35, 92D30

## Abstract

This paper presents a novel West Nile virus model that has more extensive free boundary conditions and also takes into account the impact of infected mosquitoes on the free boundary, both of which are firsts in West Nile virus modeling. Specifically, the free boundary conditions independent of the dispersal kernel functions in the equations, bring new challenges to the dynamical analysis of spreading-vanishing, especially for the case where the basic reproduction number $\mathcal R_0\le 1$, which involves new ideas and techniques for dynamics analysis. Moreover, due to the consideration of the impact of infected mosquitoes in the free boundary conditions, new conclusions have been obtained. Numerical schemes have been developed, which not only verify qualitative theoretical results, but also provide novel quantitative insights into the effects of various factors on transmission dynamics. Overall, our results not only differ significantly from the local diffusion version presented in Lin and Zhu (2017) but also extend all the conclusions from the nonlocal diffusion version in Du and Ni (2020), with some conclusions obtained under more general conditions.

## Introduction

West Nile virus (WNv) is a mosquito-borne virus of the Flaviviridae family, which was first isolated from the blood of a febrile woman in the West Nile province of Uganda in 1937 (Abdelrazec et al. 2014; Bowman et al. 2005), and first brought a major outbreak in New York City in late summer of 1999 and spread rapidly across North America (Thomas and Urena 2001; Lin and Zhu 2017; Moschini et al. 2017; Ben-Nathan et al. 2006; Abdelrazec et al. 2014; Wonham et al. 2004). The transmission of the virus circulates between mosquitoes and birds, with humans and other vertebrates being incidental hosts (Ben-Nathan et al. 2006; Abdelrazec et al. 2014; Wonham et al. 2004). It’s worth noting that the virus is transmitted to humans by mosquitoes, but cannot be transmitted back to mosquitoes (Thomas and Urena 2001; Abdelrazec et al. 2014). Therefore, humans are a dead-end host in the life cycle of the virus. Unfortunately, humans exhibit adverse effects from the virus (Bowman et al. 2005). The virus can cause inflammation of the brain and usually results in flu-like symptoms such as fever, vomiting, and stiffness of the neck (Ceausu et al. 1997; Bowman et al. 2005). The disease can lead to severe symptoms, including altered consciousness, extremity tremors, paralysis, and death (Tsai et al. 1998; Bowman et al. 2005). Since 1999, there have been more than 18,698 reported cases of WNv in humans, with more than 715 deaths (Ben-Nathan et al. 2006). There is no specific treatment available for the virus and therefore, control of the disease depends on breaking the transmission cycle of the virus (Thomas and Urena 2001; Bowman et al. 2005). These findings underscore the importance of understanding the transmission mechanisms of WNv (Lin and Zhu 2017; Thomas and Urena 2001).

To the best of our knowledge, the first model of WNv was proposed in Thomas and Urena (2001), which is a difference equation model. Subsequently, studies (Wonham et al. 2004; Bowman et al. 2005; Abdelrazec et al. 2014) used systems of ordinary differential equations (ODEs) to model the spread of WNv in mosquito and bird populations. To describe the spatio-temporal transmission dynamics, random diffusion terms are included in the models to approximate the movement of birds and mosquitoes. For instance, the study in Lewis et al. (2006) proposed the following simplified model for WNv:

1 $$\begin{aligned} \begin{aligned} B_t=d_1 B_{xx}+a_1(e_1-B)M-b_1B,&\ x\in \mathbb {R}, \ t>0,\\ M_t=d_2 M_{xx}+a_2(e_2-M)B-b_2M,&\ x\in \mathbb {R}, \ t>0, \end{aligned} \end{aligned}$$

where

2 $$\begin{aligned} a_1=\frac{\alpha _B\beta _R}{e_1} \quad \text {and} \quad a_2=\frac{\alpha _M\beta _R}{e_1}, \end{aligned}$$

*B*(*x*, *t*) and *M*(*x*, *t*) are the densities of the infected bird (host) and mosquito (vector) populations at position *x* and time *t*, respectively. The parameters are all positive constants: $d_1$ and $d_2$ are the diffusion rates of birds and mosquitoes respectively; $b_1$ is the combination of two rates, reversion rate from infectivity to susceptibility and natural death rate of birds; $b_2$ is the mosquito death rate; $e_1$ is the total number of susceptible and infected birds, $e_2$ is the total number of susceptible and infected mosquitoes; $\alpha _B$, $\alpha _M$ respectively stand for the WNv transmission probability per bite to birds and mosquitoes; $\beta _R$ is the per-capita biting rate of mosquitoes on birds. The model is based on ideal assumptions such as that infection does not confer immunity to birds, we refer to Lewis et al. (2006) and Lin and Zhu (2017) for further details for model formulation.

Please note that the ODE version of (1) is widely known as the classical Ross-Macdonald model (Wang et al. 2020), which was formulated to describe the transmission of malaria, and is widely used to describe many other mosquito-borne diseases. Therefore, mathematical results for (1) and its variations discussed in this study are also applicable to the spread of other mosquito borne infections. A key index to characterize the transmission dynamics of (1) is the basic reproduction number, given by

3 $$\begin{aligned} \mathcal R_0=\sqrt{\frac{a_1a_2e_1e_2}{b_1b_2}}. \end{aligned}$$

It is well known that (1) admits the trivial equilibrium (0, 0), and if $\mathcal R_0>1$, then (1) has a unique positive constant equilibrium

4 $$\begin{aligned} (B^*,M^*):=\left( \frac{a_1a_2e_1e_2-b_1b_2}{a_1a_2e_2+b_1a_2},\frac{a_1a_2e_1e_2-b_1b_2}{a_1a_2e_1+a_1 b_2}\right) . \end{aligned}$$

Moreover, when the nonnegative initial function pair $(B_0, M_0) \in C(\mathbb {R}) \times C(\mathbb {R})$ has nonempty compact supports and satisfies $B_0 \le e_1$ and $M_0 \le e_2$, (1) has a unique positive solution (*B*(*t*, *x*), *M*(*t*, *x*)) defined for all $t > 0$. The main results of Lewis et al. (2006) state that

$$\left\{ \begin{array}{l} \text{ if } ~~\mathcal R_0\le 1, \text{ then } ~~(B,M) \rightarrow (0, 0) ~~ \text{ as } ~~ t\rightarrow \infty ; \\ \text{ if } ~~\mathcal R_0> 1, \text{ then } ~~(B,M) \rightarrow (B^*, M^*) ~~ \text{ as } ~~ t\rightarrow \infty . \end{array}\right. $$

When $\mathcal R_0> 1$, the convergence can be characterised by a traveling wave solution with minimal speed $c^*>0$, indicating that the virus spreads with speed $c^*$.

Although the asymptotic wave speed can approximately estimate the progressive spreading speed of the virus transmission, it does not truly reflect the spread of the virus in the early stage of the infection’s spatial expansion to a larger area. Using a free boundary to model the spatial spread of the virus can better reflect this situation. That is to say, at the forefront of the boundary of the infected area, the virus expands and moves forward, thereby triggering further spatial propagation, until the entire region or area becomes endemic. Recently, Lin and Zhu (2017), inspired by the work in Du and Lin (2010), studied the following WNv model with local diffusion and free boundaries:

5 $$\begin{aligned} {\left\{ \begin{array}{ll} B_t=d_1 B_{xx}+a_1(e_1-B)M-b_1B,& g(t)<x<h(t),\quad t>0,\\ M_t=d_2 M_{xx}+a_2(e_2-M)B-b_2M,& g(t)<x<h(t),\quad t>0,\\ B(x,t)=M(x,t)=0,& x\in \{g(t), h(t)\},\quad t>0,\\ h(0)=h_0,\quad h'(t)=-\mu B_{x}(h(t),t),& t>0,\\ g(0)=-h_0,\quad g'(t)=-\mu B_{x}(g(t),t),& t>0,\\ B(x,0)=B_{0}(x),\quad M(x,0)=M_{0}(x),& -h_0\le x\le h_0, \end{array}\right. } \end{aligned}$$

where $x=h(t)$ and $x=g(t)$ are the moving boundaries; $\mu$ is a positive constant, and the initial functions satisfy

$$\begin{aligned}\left\{ \begin{aligned}&B_0\in C^2[-h_0,h_0],\quad B_0(-h_0)=B_0(h_0)=0, \quad 0< B_0(x)\le e_1 &  \text{ in } (-h_0,h_0),\\&M_0\in C^2[-h_0,h_0],\quad M_0(-h_0)=M_0(h_0)=0, \quad 0< M_0(x)\le e_2 &  \text{ in } (-h_0,h_0). \end{aligned}\right. \end{aligned}$$

In (5), they assume that the population range of infected birds and mosquitoes is a moving interval (*g*(*t*), *h*(*t*)) and

6 $$\begin{aligned} B(t, x)=M(t, x) = 0 \quad \text{ for } ~~~~ x \in \mathbb R\setminus [g(t), h(t)]. \end{aligned}$$

The expansion rate of the range boundary is proportional to the gradient of the infected bird population density at the range boundary, i.e., $h'(t)=-\mu B_x(h(t),t)$ and $g'(t)=-\mu B_{x}(g(t),t)$. This assumption originates from the classical Stefan condition (Caffarelli and Salsa 2005; Du and Lin 2010). It is proved in Lin and Zhu (2017) that (5) has a unique solution which is defined for all $t>0$, and

$$\left\{ \begin{array}{l} \text{ if } ~\mathcal R_0\le 1, \mathrm{ then } {\boldsymbol{Vanishing}} happens: \\ \ \ \ \ \ \ ~h_\infty -g_\infty <\infty ~and \lim \limits _{t\rightarrow \infty }(\Vert B(\cdot ,t) \Vert _{C([g(t),h(t)])} +\Vert M(\cdot ,t)\Vert _{C([g(t),h(t)])})=0; \\ \text{ if } ~\mathcal {R}_0^F(t_0)\ge 1 ~~\text{ for } \text{ some }~~t_0\ge 0, \text{ then }  {\textbf {Spreading}} happens: \\ \ \ \ \ \ \ \ \ ~h_\infty =-g_\infty =\infty ~and \,\, \lim \limits _{t\rightarrow \infty }(B(x,t),M(x,t))=(B^*, M^*) \text{ uniformly } \\ \ \ \ \ \ \ \ \ \text{ in } \text{ any } \text{ bounded } \text{ set } \text{ of } \mathbb R; \\ \text{ if } ~\mathcal R_0> 1\ge \mathcal {R}_0^F(0), \text{ then } \text{ either } \text{ Spreading } \text{ happens } \text{ or } \text{ Vanishing } \text{ happens, } \end{array}\right. $$

where $\mathcal {R}_0^F(t)$ is the spatial-temporal risk index, $h_\infty :=\lim \limits _{t\rightarrow \infty }h(t)$ and $g_\infty :=\lim \limits _{t\rightarrow \infty }g(t)$. Furthermore, when $\mathcal R_0> 1\ge \mathcal {R}_0^F(0)$, the spreading or vanishing of the virus depends on the initial number of infected individuals, the area of the infected region, the diffusion rate and other factors. Recently, Wang et al. (2019) estimated the spreading speed of (5) when spreading happens.

Noting that in both (1) and (5), the spatial movement of individuals is described by the local diffusion term $du_{xx}$, which fails to account for nonlocal factors such as long-distance dispersal that are commonly observed in many real-world propagation processes (Clobert et al. 2012; Nathan et al. 2012). To incorporate such nonlocal effects, one approach is to replace $du_{xx}$ with $d\Big [\displaystyle \int _{\mathbb R}J(x-y)u(t,y){\textrm{d}}y-u(t,x)\Big ]$. This modification implies that an individual located at *x* can migrate to any other location *y* with a probability given by $J(x-y)$, and the migration frequency is *d* per unit time. In recent years, nonlocal counterparts of (1) have attracted extensive attention (see Bao et al. (2016); Bao and Shen (2017); Berestycki et al. (2016a, 2016b); Garnier (2011); Hutson et al. (2003); Li et al. (2010) and references therein). In Du and Ni (2020), a nonlocal version of the free boundary model with nonlocal diffusion was proposed and analyzed:

7 $$\begin{aligned} {\left\{ \begin{array}{ll} \displaystyle B_t=d_1 \int _{g(t)}^{h(t)}J_1(x-y)B(t,y)\textrm{d}y-d_1 B(t,x) \\ ~~~~~~~~\displaystyle +a_1(e_1-B(t,x))M(t,x)-b_1 B(t,x), & x\in (g(t), h(t)),\ t>0,\\ \displaystyle M_t=d_2 \int _{g(t)}^{h(t)}J_2(x-y)M(t,y)\textrm{d}y-d_2 M(t,x) \\ ~~~~~~~~+a_2(e_2-M(t,x))B(t,x)-b_2 M(t,x) , & x\in (g(t), h(t)),\ t>0,\\ B(t,x)= M(t,x)=0, & t>0, \; x\in \{g(t), h(t)\},\\ \displaystyle g'(t)= -\mu \int _{g(t)}^{h(t)}\int _{-\infty }^{g (t)}J_1(x-y)B(t,x)\textrm{d}y\textrm{d}x, & t>0,\\ \displaystyle h'(t)= \mu \int _{g(t)}^{h(t)}\int _{h(t)}^{\infty }J_1(x-y)B(t,x)\textrm{d}y\textrm{d}x, & t >0,\\ B(0,x)=u_{10}(x),\ M(0,x)=u_{20}(x), \  & x\in [-h_0,h_0], \end{array}\right. } \end{aligned}$$

where

$$\begin{aligned} \int _{g(t)}^{h(t)}J_1(x-y)B(t,y)\textrm{d}y=\int _{\mathbb {R}}J_1(x-y)B(t,y)\textrm{d}y, \end{aligned}$$

$$\begin{aligned} \text {and } \int _{g(t)}^{h(t)}J_2(x-y)M(t,y)\textrm{d}y=\int _{\mathbb {R}}J_2(x-y)M(t,y)\textrm{d}y \end{aligned}$$

since they also assume (6) holds, and the initial functions $u_{i0}(x)$ $(i=1,2)$ satisfy

8 $$\begin{aligned} &  u_{i0}\in C([-h_0,h_0]),~ u_{i0}(-h_0)=u_{i0}(h_0)=0,~\nonumber \\ &  \quad 0<u_{i0}(x)\le e_i\ \textrm{for}\ x\in (-h_0,h_0), i=1,2. \end{aligned}$$

The kernel functions $J_i:{\mathbb R}\rightarrow {\mathbb R}$ $(i=1,2)$ satisfy

$(\textbf{J})$: $J_i\in C({\mathbb R})\cap L^\infty ({\mathbb R})$ is nonnegative, symmetric, $J_i(0)>0$, $\displaystyle \int _{\mathbb R}J_i(x) \textrm{d}x=1$, *i*=1,2.

The results in Du and Ni (2020) show that if $(\textbf{J})$ and (8) hold, then problem (7) admits a unique positive solution (*B*, *M*, *g*, *h*) defined for all $t>0$ and

$$\left\{ \begin{array}{l} \text{ if } ~\mathcal R_0\le 1, \mathrm{ then } {\boldsymbol{Vanishing}} happens: \\ \ \ \ ~h_\infty -g_\infty <\infty ~and \lim \limits _{t\rightarrow \infty }(\Vert B(\cdot ,t) \Vert _{C([g(t),h(t)])} +\Vert M(\cdot ,t)\Vert _{C([g(t),h(t)])})=0; \\ \text{ if } ~\mathcal R_0> 1, \text{ then } \text{ either } \text{ Vanishing } \text{ happens } \mathrm{ or } {\boldsymbol{Spreading}}~happens: \\ \ \ \ \ ~h_\infty =-g_\infty =\infty ~and \lim \limits _{t\rightarrow \infty }(B(x,t),M(x,t))=(B^*, M^*) \text{ uniformly } \\ \ \ \ \ \text{ in } \text{ any } \text{ bounded } \text{ set } \text{ of } {\mathbb R.} \end{array}\right. $$

Moreover, they also provided specific criteria for the case where $\mathcal R_0>1$, which depends on $u_{i0}$, $h_0$, and other parameters. Different from random diffusion model (5), the nonlocal diffusion variation (7) leads to the emergence of accelerated diffusion phenomenon. In addition, Du and Ni (2022) also presented the estimation of the finite propagation speed.

It is not difficult to find that if we define

9 $$\begin{aligned} K_{J_1}(x):=\int _{x}^{+\infty } J_1(y) \textrm{d} y, \end{aligned}$$

then the free boundary conditions in (7) can be written equivalently as

10 $$\begin{aligned} {\left\{ \begin{array}{ll} \displaystyle h^{\prime }(t) =\mu F_{h, K_{J_1}}:=\mu \int _{g(t)}^{h(t)} B(t, x) K_{J_1}(h(t)-x) \textrm{d} x,\\ \displaystyle g'(t) =-\mu F_{g, K_{J_1}}:=-\mu \int _{g(t)}^{h(t)} B(t, x) K_{J_1}(x-g(t)) \textrm{d} x. \end{array}\right. } \end{aligned}$$

As shown in Cao et al. (2019), $F_{h, K_{J_1}}$ is the outward flux of a population at free boundary *h*(*t*), that is, the total population mass moved out of the range [*g*(*t*), *h*(*t*)] at time *t* through its right boundary $x = h(t)$ per unit time. Furthermore, $h^{\prime }(t) =\mu F_{h, K_{J_1}}$ can be interpreted as assuming that the expanding rate of the front is proportional to the outward flux. And $F_{g, K_{J_1}}$ can also be explained in a similar manner. Obviously, (10) indicates that the outward flux is closely dependent on the diffusion kernel function $J_1$, and the pushing of the free boundary is only influenced by infected birds.

A natural question arises: what happens when the outward flux is determined by a general function *K* instead of $K_{J_1}$? In fact, a recent study (Long et al. 2024) considered this issue and proposed the following new free boundary conditions:

11 $$\begin{aligned} {\left\{ \begin{array}{ll} \displaystyle h^{\prime }(t) =\mu F_{h, K}:=\mu \int _{g(t)}^{h(t)} B(t, x) K(h(t)-x) \textrm{d} x,\\ \displaystyle g'(t) =-\mu F_{g, K}:=-\mu \int _{g(t)}^{h(t)} B(t, x) K(x-g(t)) \textrm{d} x, \end{array}\right. } \end{aligned}$$

which indicates that the contribution of each point in [*g*(*t*), *h*(*t*)] to the outward flux at *h*(*t*) is closely related to a general weighting function *K* of the distance from that point to the boundary *h*(*t*). This represents a very different assumption that the movement of species boundaries can be independent of their dispersal strategies. Such a function $K(h(t)-x)$ has also been applied in bird flight models to describe that the movement of individuals within a population is restricted by their distance from the boundary (Nagy et al. 2010). For example, *K* may be chosen as a combination of certain exponential functions (Feng et al. 2022). In general, we can find that the free boundary conditions in some existing studies (Cao et al. (2019); Du et al. (2021, 2022); Lin and Zhu (2017); Wang et al. (2019); Du and Ni (2020, 2022); Du et al. (2024)) are a special case of (11), which represents a more extensive free boundary condition.

On the other hand, both (5) and (7) assume that the expansion speed of the infection boundary front is only driven by the infected birds (Du and Ni 2020; Lin and Zhu 2017). This is because they assumed the impact of mosquitoes on the progression of the infection boundary front to be negligible. However, since the number of mosquitoes far exceeds that of birds and mosquitoes move freely, they may have a significant capacity to spread viruses and push the free boundary. Therefore, it would be more reasonable to consider that both infected mosquitoes and birds play a role in advancing the infection frontier.

Inspired by the above discussion, in this paper, we study the following model:

12 $$\begin{aligned} {\left\{ \begin{array}{ll} \displaystyle B_t=d_1 \int _{g(t)}^{h(t)}J_1(x-y)B(t,y)\textrm{d}y-d_1 B(t,x) \\ ~~~~~~~~~~~~+a_1(e_1-B(t,x))M(t,x)-b_1 B(t,x), & t>0,\ x\in (g(t), h(t)),\\ \displaystyle M_t=d_2 \int _{g(t)}^{h(t)}J_2(x-y)M(t,y)\textrm{d}y-d_2 M(t,x) \\ ~~~~~~~~~~~~+a_2(e_2-M(t,x))B(t,x)-b_2 M(t,x) , & t>0,\ x\in (g(t), h(t)),\\ \displaystyle g'(t)= -\mu _1 \int _{g(t)}^{h(t)}K_1(x-g(t))B(t,x)\textrm{d}x \\ ~~~~~~~~~~~~ \displaystyle -\mu _2 \int _{g(t)}^{h(t)}K_2(x-g(t))M(t,x)\textrm{d}x, & t>0,\\ \displaystyle h'(t)= \mu _1 \int _{g(t)}^{h(t)}K_1(h(t)-x)B(t,x)\textrm{d}x \\ ~~~~~~~~~~~~ \displaystyle + \mu _2 \int _{g(t)}^{h(t)}K_2(h(t)-x)M(t,x)\textrm{d}x, & t>0,\\ B(t,x)= M(t,x)=0, & t >0, \; x\in \{g(t), h(t)\},\\ B(0,x)=u_{10}(x),\ M(0,x)=u_{20}(x), \  & x\in [-h_0,h_0], \end{array}\right. } \end{aligned}$$

where constants $\mu _1,\mu _2>0$ and the initial functions $u_{i0}(x)$ $(i=1,2)$ satisfy (8). We assume infection does not confer immunity to birds, the kernel functions $J_i:{\mathbb R}\rightarrow {\mathbb R}$ $(i=1,2)$ satisfy $(\textbf{J})$, and $K_i$ $(i=1,2)$ satisfy the following basic condition

***(K)***: $$K_i(x)$$ are nonnegative and locally Lipschitz continuous in $$[0,\infty )$$, and $$K_i (0)>0 $$.

Sometimes we also require $K_i$ to satisfy the additional condition

***(K1)***: For any $y\in [0,\infty )$, there exist constants $\kappa _i>0$ such that $\displaystyle \int _{y}^{\infty } J_i(x) {\textrm{d}}x\ge \kappa _i K_i(y)$, $i=1,2$.

The main results in this study can be summarized in the following three theorems.

### Theorem 1.1

(Existence and uniqueness) Assume that $(\textbf{J})$ and $(\textbf{K})$ hold. Then for any given $h_0>0$ and $(u_{10},u_{20} )$ satisfy (8), problem (12) admits a unique positive solution (*B*, *M*, *g*, *h*) defined for all $t>0$.

### Theorem 1.2

(Spreading-vanishing dichotomy) Assume $(\textbf{J})$, $(\textbf{K})$ and $(\textbf{K1})$ hold, and the initial functions satisfy (8). Let (*B*, *M*, *g*, *h*) be the solution of (12), and denote

13 $$\begin{aligned} g_\infty :=\lim \limits _{t\rightarrow \infty }g(t) \ \ \ \ \textrm{and} \ \ \ \ h_\infty :=\lim \limits _{t\rightarrow \infty }h(t). \end{aligned}$$

Then one of the following alternatives must occur:

(i) **Spreading**: $$-g_\infty =h_\infty =\infty $$ and
  $$\begin{aligned} \lim \limits _{t\rightarrow \infty }(B(t,x), M(t,x))=(B^*,M^*) \text{ locally } \text{ uniformly } \text{ in } {\mathbb R}; \end{aligned}$$
(ii) **Vanishing**: $h_\infty -g_\infty <\infty$ and
  $$ \lim \limits _{t\rightarrow \infty }(B(t,x), M(t,x))=(0,0) \text{ uniformly } \text{ for } x\in [g(t),h(t)]. $$

### Theorem 1.3

(Spreading-vanishing criteria) Assume $(\textbf{J})$ and $(\textbf{K})$ hold, and the initial functions satisfy (8). Let (*B*, *M*, *g*, *h*) be the solution of (12), and $\mathcal R_0$ be given by (3).

(i) If $\mathcal R_0<1$ and $\limsup \limits _{y\rightarrow \infty }\frac{\int _0^y K_i(x)\textrm{d}x}{y}<\infty ~(i=1,2)$, then vanishing always happens.
(ii) If $\mathcal R_0=1$ and $(\textbf{K1})$ holds, then vanishing always happens.
(iii) If $\mathcal R_0>1$ and one of the following conditions holds
  $$\begin{aligned}&1.\ \ {\frac{a_1a_2e_1e_2}{(b_1+d_1)(b_2+d_2)}} \ge 1, \\&2.\ \ {\frac{a_1a_2e_1e_2}{(b_1+d_1)(b_2+d_2)}}<1,\ \ h_0\ge L^*, \end{aligned}$$
  then spreading always happens, where $L^*$ is a fixed constant depending on $(a_i, b_i, d_i, e_i, J_i)$ $(i=1,2)$.
(iv) If $\mathcal R_0>1$ and
  $$\begin{aligned}&{\frac{a_1a_2e_1e_2}{(b_1+d_1)(b_2+d_2)}}<1,\ \ h_0<L^*, \end{aligned}$$
  then the following conclusions hold:
  - For any fixed $\mu _1+\mu _2>0$ and sufficient small initial datum $(u_{10},u_{20} )$, vanishing happens.
  - For any fixed $\mu _2>0$ and any given initial function pair $(u_{10},u_{20} )$ satisfying (8), there exists $\mu ^*:=\mu ^*(u_{10},u_{20} )>0$ such that vanishing happens for $0<\mu _1+\mu _2\le \mu ^*$ and spreading happens for $\mu _1+\mu _2>\mu ^*$.
  - For any fixed $\mu _1>0$ and any given initial function pair $(u_{10},u_{20} )$ satisfying (8), there exists $\mu _*:=\mu _*(u_{10},u_{20} )>0$ such that vanishing happens for $0<\mu _1+\mu _2\le \mu _*$ and spreading happens for $\mu _1+\mu _2>\mu _*$.

### Remark 1.4

In fact, for the existing free boundary conditions considered in Cao et al. (2019) and Du and Ni (2020), condition $(\textbf{K1})$ is automatically satisfied, as in these models the boundary kernel is given by $K_i(x) = \int _x^{+\infty } J_i(y)\,dy$. This observation indicates that condition $(\textbf{K1})$ serves as a natural generalization of the assumption adopted in the earlier models.

From a biological perspective, the boundary kernel function $K_i$ characterizes the capacity of a population to expand outward at the moving boundary. In addition to the dispersal strategy within the habitat, as described by the kernel $J_i$, this expansion capacity is also modulated by external factors such as environmental resistance and natural predation. Condition $(\textbf{K1})$ thus imposes a biologically reasonable constraint that links the mechanism of boundary expansion to the internal dispersal process: although the expansion capacity at the boundary may differ from the dispersal strategy within the habitat, it remains regulated by the latter and cannot exceed the dispersal kernel functions $J_i$ by an unreasonable margin.

Before moving to the theoretical proofs for these results in the next section, we would like to briefly highlight main contributions of the current study.

### Remark 1.5

Some remarks on the current study:

(i) **[Model formulation:]** Model (12) incorporates more extensive free boundary conditions independent of dispersal kernel functions and includes the impact of infected mosquitoes on boundary expansion. To our knowledge, these aspects are novel in the WNv modeling.
(ii) **[Theoretical challenges:]** The introduction of new free boundary conditions, independent of the dispersal kernel $J_i$, involves novel ideas and techniques for dynamics analysis. For instance, we construct a new upper solution for the case $\mathcal {R}_0 < 1$.
(iii) **[Extending existing results:]** By selecting appropriate $K_i$ ($i=1,2$), (7) becomes a special case of model (12). Our conclusions extend those in Du and Ni (2020), as shown in Theorem 1.3-(i,iii,iv), to general conditions of $K_i$. The introduction of the parameter $\mu _2$ also leads to new findings.
(iv) **[Distinct results:]** Compared to the local diffusion model (5), the nonlocal diffusion model (12) yields distinct results. For example, Theorem 1.3-(iii) shows that spreading occurs regardless of $h_0$ and $\mu$ when $\mathcal {R}_0 > 1$ and $d_1, d_2 \ll 1$, contrasting sharply with (5) (see Lin and Zhu 2017, Theorem 5.5).
(v) **[Numerical schemes:]** We developed numerical schemes to solve this nonlocal diffusion model with free boundaries defined by nonlocal integrals. Simulations verify the reliability of our conclusions and provide quantitative insights. To our knowledge, this appears to be the first numerical work for such extensive free boundary conditions, where $K_i(i=1,2)$ is merely a general non negative locally Lipschitz continuous function.

As in some existing studies (Du et al. (2021); Du and Ni (2022); Wang et al. (2019)), when propagation occurs, one naturally wonders what would be the spreading speed. This question will be explored in a subsequent paper. The rest of this paper is organised as follows. Section 2 introduces some notations and presents preliminary results on the comparison principle, the associated eigenvalue problem, and the fixed boundary problem, which are crucial for our later analysis. Section 3 establishes the global existence and uniqueness of solutions for a more general system, of which both (7) and (12) are a special case. The conditions needed are weaker than those in Du et al. (2022). Section 4 is dedicated to proving Theorems 1.2 and 1.3, which extensively utilizes the results from Sect. 2, and requires new ideas and techniques to address the challenges posed by the new free boundary conditions independent of *J*. This is particularly evident in addressing the scenario where $\mathcal {R}_0 \le 1$ and managing the introduction of the new parameter $\mu _2$. Sect. 5 focuses on numerical simulations to verify theoretical results and provide additional quantitative insights.

## Some preparations

This section is going to introduce some notations and establish basic results for the convenience of later use and reference. For given $-g_0$, $h_0$, $T>0$, we define

$$\begin{aligned}&\mathbb G_{T}=\mathbb G_{g_0,T}:=\{g\in C([0,T]): g(0)=g_0,\ \sup _{0\le t_1<t_2\le T}\frac{g(t_2)-g(t_1)}{t_2-t_1}<0\},\\&\mathbb H_{T}=\mathbb H_{h_0,T}:=\{h\in C([0,T]): h(0)=h_0,\ \inf _{0\le t_1<t_2\le T}\frac{h(t_2)-h(t_1)}{t_2-t_1}>0 \}. \end{aligned}$$

For any given $g\in \mathbb G_{T}$, $h\in \mathbb H_{T}$ and

14 $$\begin{aligned} u_{i0}\in C([g_0,h_0]),\ \ u_{i0}(g_0)=u_{i0}(h_0)=0,\ u_{i0}(x)>0\ \text{ in } (g_0,h_0),\; i=1,2, \end{aligned}$$

set

$$\begin{aligned}&\Delta _T=\Delta _T^{g,h}:=\{(t,x): t\in (0,T],\; g(t)<x<h(t))\},\\&\mathbb X_{T}=\mathbb X_{T}^{g,h}:=\{(\phi _1,\phi _2): \phi _i\in C(\overline{\Delta }_T),\; \phi _i\ge 0,\ \ \phi _i(0,x)=u_{i0}(x)\ \textrm{in}\ [g_0,h_0], \\&\qquad \textrm{and} \ \phi _i(t,g(t))=\phi _i(t,h(t))=0\ \textrm{in}\ [0,T],\; i=1,2\}. \end{aligned}$$

Thanks to Lemma 3.1 in Du and Ni (2020), we can directly obtain the following results.

### Lemma 2.1

(Maximum principle) Assume that **(J)** holds and $(g,h)\in \mathbb G_{ T} \times \mathbb H_{T}$ for given $-g_0, h_0, T>0$. If $\phi _i$, $\partial _t\phi _i\in C(\overline{\Delta }_T)$, $d_i$, $c_{ij}\in L^\infty (\Delta _T)$, $d_i\ge 0$, $i=1,2$, and

15 $$\begin{aligned} {\left\{ \begin{array}{ll} \displaystyle \partial _t\phi _i\ge d_i\int _{g(t)}^{h(t)}J_i(x-y)\phi _i(t,y)\textrm{d}y-d_i\phi _i(t,x)+\sum _{j=1}^n c_{ij}\phi _j, & (t,x)\in \Delta _T,\; \\ \phi _i(t,g(t))\ge 0, \phi _i(t,h(t))\ge 0, & t \in (0, T],\; \\ \phi _i(0,x)\ge 0, & x\in [g_0,h_0]. \end{array}\right. } \end{aligned}$$

Then the following conclusions hold:

(i) If $c_{ij}\ge 0$ on $\Delta _T$ for $i, j\in \{1,\cdots , n\}$ and $i\not =j$, then $\phi _i\ge 0$ on $\overline{\Delta }_T$ for $i\in \{1,\cdots , n\}$.
(ii) If in addition $d_{i_0}>0$ in $\Delta _T$, $\phi _{i_0}(0,x)\not \equiv 0$ in $[g_0,h_0]$, then $\phi _{i_0}> 0$ in $\Delta _T$.

### Remark 2.2

When *g*(*t*) and *h*(*t*) are constants, the conclusion of Lemma 2.1 still holds without requiring the second line of (15). For details, see Lemma 3.1 in Du and Ni (2020).

For any $L>0$, the following results hold for the associated eigenvalue problem:

16 $$\begin{aligned} {\left\{ \begin{array}{ll} \displaystyle -d_1 \int _{-L}^{L}J_1(x-y)\phi (y)\textrm{d}y-d_1 \phi (x) =a_1e_1\psi -b_1 \phi +\lambda \phi , & x\in [-L, L],\\ \displaystyle -d_2 \int _{-L}^{L}J_2(x-y)\psi (y)\textrm{d}y-d_2 \psi (x)=a_2e_2\phi -b_2 \psi +\lambda \psi , & x\in [-L, L]. \end{array}\right. } \end{aligned}$$

### Proposition 2.3

(Bao and Shen, 2017, Theorems 2.2 and 2.3) Assume $(\textbf{J})$ holds. Then (16) has a principal eigenvalue $\lambda =\lambda _1(-L,L)$ with a positive eigenfunction pair $(\phi , \psi )=(\phi _1,\psi _1)\in C([-L,L])\times C([-L, L])$. Moreover, $\lambda _1(-L,L)$ is an algebraically simple eigenvalue.

### Proposition 2.4

(Du and Ni, 2020, Corollary 2.5) Assume $(\textbf{J})$ holds. Let $l_1<l_2$, and $\lambda _1(l_1,l_2)$ be the principal eigenvalue of (16) with $[-L,L]$ replaced by $[l_1,l_2]$. Then

(i) $\lambda _1(l_1,l_2)$ is strictly decreasing with respect to $l_2-l_1$, and is continuous in $l_1$ and $l_2$.
(ii) If $\mathcal { R}_0\le 1$, then $\lambda _1(l_1,l_2)>0$ for any $l_1$ and $l_2$.
(iii) If $\mathcal { R}_0>1$ and
  $$\begin{aligned} {\frac{a_1a_2e_1e_2}{(d_1+b_1)(d_2+b_2)}}\ge 1, \end{aligned}$$
  then $\lambda _1(l_1,l_2)<0$ for any $l_1$ and $l_2$.
(iv) If $\mathcal { R}_0>1$ and
  $$\begin{aligned} {\frac{a_1a_2e_1e_2}{(d_1+b_1)(d_2+b_2)}}<1, \end{aligned}$$
  then there exists $L^*>0$ such that $\lambda _1(l_1,l_2)=0$ for $l_2-l_1=2L^*$, and
  $$\begin{aligned} \lambda _1(l_1,l_2)> 0\ (\mathrm{resp.}<0) \quad \textrm{for} \quad l_2-l_1<2L^*\ (\mathrm{resp.}\ l_2-l_1>2L^*). \end{aligned}$$

For any $L>0$, we define $Q_L=(0, \infty )\times (-L,L)$ and consider the corresponding fixed boundary problem of (12):

17 $$\begin{aligned} {\left\{ \begin{array}{ll} \displaystyle B_t=d_1 \int _{-L}^{L}J_1(x-y)B(t,y)\textrm{d}y-d_1B(t,x)+a_1(e_1-B)M-b_1B, & (t,x)\in Q_L,\\ \displaystyle M_t=d_2 \int _{-L}^{L}J_2(x-y)M(t,y)\textrm{d}y-d_2M(t,x)+a_2(e_2-M)B-b_2 M, & (t,x)\in Q_L,\\ B(0,x)=B_0(x), M(0,x) =M_0(x),& x\in [-L,L], \end{array}\right. } \end{aligned}$$

where $B_0$, $M_0\in C([-L,L])\setminus \{0\}$, and $0\le B_0\le e_1,\; 0\le M_0\le e_2$. It is well-known that (17) has a unique positive solution which is defined for all $t>0$. Clearly, the corresponding steady state problem of (17) is

18 $$\begin{aligned} {\left\{ \begin{array}{ll} \displaystyle d_1 \int _{-L}^{L}J_1(x-y)\widetilde{B}(y)\textrm{d}y-d_1\widetilde{B}(x){+}a_1(e_1-\widetilde{B})\widetilde{M}-b_1 \widetilde{B}{=}0, & x\in [-L,L],\\ \displaystyle d_2 \int _{-L}^{L}J_2(x-y)\widetilde{M}(y)\textrm{d}y-d_2\widetilde{M}(x){+}a_2(e_2-\widetilde{M})\widetilde{B}-b_2 \widetilde{M}{=}0, & x\in [-L,L]. \end{array}\right. } \end{aligned}$$

### Proposition 2.5

(Du and Ni, 2020, Proposition 3.4 and 3.5) Assume $(\textbf{J})$ holds and (*B*, *M*) is the unique positive solution of (17). Then we have the following conclusions.

(i) The problem (17) has a unique positive steady state $(\widetilde{B},\widetilde{M})\in C([-L,L])\times C([-L, L])$ satisfying $0<\tilde{B}\le e_1,\; 0<\tilde{M}\le e_2$ if $\lambda _1(-L,L)<0$, and (0, 0) is the only nonnegative steady-state when $\lambda _1(-L,L)\ge 0$, where $\lambda _1(-L,L)$ is the principal eigenvalue of (16).
(ii) If $\lambda _1(-L,L)\ge 0$, then (*B*, *M*) converges to (0, 0) as $t\rightarrow \infty$ uniformly for $x\in [-L, L]$.
(iii) If $\lambda _1(-L,L)< 0$, then (*B*, *M*) converges to $(\widetilde{B},\widetilde{M})$ as $t\rightarrow \infty$ uniformly for $x\in [-L, L]$.
(iv) If $\mathcal { R}_0>1$, then $\lambda _1(-L,L)< 0$ for all large *L*, and $\lim \limits _{L\rightarrow \infty }(\widetilde{B}_L(x),\widetilde{M}_L(x))=(B^*,M^*)$ locally uniformly in $\mathbb {R}$, where $(B^*,M^*)$ is defined by (4) and $(\widetilde{B}_L, \widetilde{M}_L):=(\widetilde{B}, \widetilde{M})$ in order to stress its dependence on *L*.

## Global existence and uniqueness of solutions

This section establishes the global existence and uniqueness of the solutions for the problem (12). We will directly consider a more general model as follows:

19 $$\begin{aligned} {\left\{ \begin{array}{ll} \displaystyle \partial _tu_i{=}d_i \int _{g(t)}^{h(t)}J_i(x-y)u_i(t,y)\textrm{d}y{-}d_iu_i{+}f_i(t,x,u_1,u_2), & t\in (0, T], x\in (g(t), h(t)) ,\\ u_i(t,g(t))= u_i(t,h(t))=0, & t \in (0, T],\\ \displaystyle g'(t)= -\sum _{i=1}^2\mu _i \int _{g(t)}^{h(t)}K_i(x-g(t))u_i(t,x)\textrm{d}x, & t \in (0, T],\\ \displaystyle h'(t)= \sum _{i=1}^2\mu _i \int _{g(t)}^{h(t)}K_i(h(t)-x)u_i(t,x)\textrm{d}x, & t \in (0, T],\\ u_i(0,x)=u_{i0}(x), ~~i=1,2, & x\in [g_0,h_0], \end{array}\right. } \end{aligned}$$

where constants $-g_0, h_0>0$, $\mu _1\ge 0$, $\mu _2\ge 0$ with $\mu _1+\mu _2>0$, and the initial values satisfy (14). Let ${\mathbb R}^+:=[0,\infty )$, and assume that functions $f_i:{\mathbb R}^+\times {\mathbb R}\times {\mathbb R}^+\times {\mathbb R}^+\rightarrow {\mathbb R}$ $(i=1,2)$ satisfy

$\mathbf {(F)}$: $f_1(t,x,0,u_2)\ge 0$, $f_2(t,x,u_1,0)\ge 0$ and for $i\in \{1,2\}$, $f_i(t,x,0,0)\equiv 0$, $f_i(t,x,u_1,u_2)\in C({\mathbb R}^+\times {\mathbb R}\times {\mathbb R}^+\times {\mathbb R}^+)$ and is locally Lipschitz continuous in $(u_1,u_2)$ uniformly in (*t*, *x*), i.e., for any $\rho _1$, $\rho _2>0$, there is a constant $L(\rho _1,\rho _2)>0$ such that
  $$\begin{aligned} |f_i(t,x,u_1,u_2)-f_i(t,x,v_1,v_2)|\le L(\rho _1,\rho _2) (|u_1-v_1|+|u_2-v_2|),\; i=1,2, \end{aligned}$$
  for $u_1,\, v_1\in [0,\rho _1]$, $u_2,\, v_2\in [0,\rho _2]$ and $(t,x)\in {\mathbb R}^+\times {\mathbb R}$. If $\rho _1=\rho _2$, then $L(\rho _1):=L(\rho _1,\rho _2)$.
$\mathbf {(F1)}$: There exists $k_0>0$ such that
  $$\begin{aligned} f_1(t,x,u_1,u_2)<0 \text{ for } (t,x,u_1,u_2)\in {\mathbb R}^+\times {\mathbb R}\times [k_0,\infty ) \times (0,\infty ). \end{aligned}$$
$\mathbf {(F2)}$: For any given $l>0$, there exists $\theta (l)>0$ such that
  $$\begin{aligned} f_2(t,x,u_1,u_2)<0 \text{ for } (t,x,u_1,u_2)\in {\mathbb R}^+\times {\mathbb R}\times (0,l]\times [\theta (l),\infty ) . \end{aligned}$$

By $\mathbf {(F)}$, we have

$$\begin{aligned} |f_i(t,x,u_1,u_2)|\le &  L(\rho _1,\rho _2) (u_1+u_2) \text{ for } (t,x,u_1,u_2)\in {\mathbb R}^+\\ &  \times {\mathbb R}\times [0,\rho _1]\times [0,\rho _2],~~i=1,2. \end{aligned}$$

Moreover, due to $f_1(t,x,0,u_2)\ge 0$, we can see that

20 $$\begin{aligned} \begin{aligned} f_1(t,x,u_1,u_2)\ge&f_1(t,x,u_1,u_2)-f_1(t,x,0,u_2) \\ \ge&-|f_1(t,x,u_1,u_2)-f_1(t,x,0,u_2)| \\ \ge&-L(\rho _1, \rho _2)u_1. \end{aligned} \end{aligned}$$

Similarly,

21 $$\begin{aligned} \begin{aligned} f_2(t,x,u_1,u_2)\ge -|f_2(t,x,u_1,u_2)-f_2(t,x,u_1,0)| \ge -L(\rho _1, \rho _2)u_2 \end{aligned} \end{aligned}$$

for $(t,x,u_1,u_2)\in {\mathbb R}^+\times {\mathbb R}\times [0,\rho _1]\times [0,\rho _2]$. It is easy to see that nonlinear functions in (12) are given by

$$\begin{aligned} f_1(u_1,u_2)=a_1(e_1-u_1)u_2-b_1u_1,\ \ f_2(u_1,u_2)=a_2(e_2-u_2)u_1-b_2 u_2, \end{aligned}$$

which satisfy the conditions $\mathbf {(F),(F1)}$ and $\mathbf {(F2)}$. In light of the inequalities $f_1(t, x, 0, u_2) > 0$ and $f_2(t, x, u_1, 0) > 0$ for $u_1, u_2 > 0$, it is evident that these functions do not satisfy the condition $\mathbf {(f)}$ in Du et al. (2022). Consequently, the conditions considered in this study are less restrictive than those presented in Du et al. (2022).

In view of Lemma 4.2 in Du and Ni (2020), we can directly obtain the following conclusions, which will be used in the proof of Theorem 1.1.

### Lemma 3.1

Assume $\mathbf {(F),(F1), (F2)}$, $(\textbf{J})$ hold and $u_{10}, u_{20}$ satisfy (14). Then, for any $(g,h)\in \mathbb G_{T}\times \mathbb H_{T}$, the following problem for variables $v_i(t,x)$ with $i=1,2$

$$\begin{aligned} {\left\{ \begin{array}{ll} \partial _tv_i=\displaystyle d_i \int _{g(t)}^{h(t)}J_i(x-y)v_i(t,y)\textrm{d}y-d_i v_i(t,x)+f_i(t,x,v_1,v_2), & (t,x)\in \Delta _T,\\ v_i(t,g(t))= v_i(t,h(t))=0, & t \in (0, T],\\ v_i(0,x)=u_{i0}(x), & x\in [g_0,h_0], \end{array}\right. } \end{aligned}$$

has a unique solution $V=(v_1^{g,h},v_2^{g,h})\in C(\overline{\Delta }_T)\times C(\overline{\Delta }_T)$, and

$$\begin{aligned}&0<v_1^{g,h}\le \max \{||u_{10}||_{\infty },k_0\}=:M_0^1&\textrm{in}\ \Delta _T,\\&0<v_2^{g,h}\le \max \{||u_{20}||_{\infty },\theta (k_0)\}=:M_0^2&\textrm{in}\ \Delta _T, \end{aligned}$$

where $k_0, \theta (k_0)$ are defined in the assumptions $(\textbf{F1})$ and $(\textbf{F2})$.

Now, for $(g,h)\in \mathbb G_{T}\times \mathbb H_{T}$, we define a mapping $\Gamma (g,h)(t):= \left( \tilde{g} (t),\,\tilde{h}(t)\right)$ by

22 $$\begin{aligned} \left\{ \begin{aligned}&\tilde{g}(t):=g_0-\sum _{i=1}^2\mu _i \int _0^t \int _{g(\tau )}^{h(\tau )} v^{g,h}_i(\tau ,x) K_i(x-g(\tau )) \textrm{d} x \textrm{d} \tau , \;\; t \in (0, T],\\&\tilde{h}(t):=h_0+\sum _{i=1}^2\mu _i \int _0^t \int _{g(\tau )}^{h(\tau )}v^{g,h}_i(\tau ,x) K_i(h(\tau )-x) \textrm{d} x\textrm{d} \tau ,\;\; t \in (0, T] . \end{aligned} \right. \end{aligned}$$

For any positive constants $c, s, \epsilon$, denote

$$\begin{aligned} \Sigma _{c,s,\epsilon }:=&\Big \{(g,h)\in \mathbb G_s\times \mathbb H_s: \sup _{0\le t_1<t_2\le s}\frac{g(t_2)-g(t_1)}{t_2-t_1}\le -c,\;\\&\hspace{0.5cm} \inf _{0\le t_1<t_2\le s}\frac{h(t_2)-h(t_1)}{t_2-t_1}\ge c, h(t)-g(t)\le h_0-g_0+{\epsilon } \text{ for } t\in [0, s]\Big \}. \end{aligned}$$

Then, $(\Sigma _{c,s,\epsilon }, d)$ is a complete metric space, where

$$ d\left( (h_1,g_1),(h_2,g_2)\right) :=\Vert h_1-h_2\Vert _{C([0,s])}+ \Vert g_1-g_2\Vert _{C([0,s])}~~~\text{ for }~~(g_1, h_1), (g_2, h_2)\in \Sigma _{c,s,\epsilon }. $$

Obviously, if $(g,h) \in \Sigma _{c,s,\epsilon }$, then

23 $$\begin{aligned} ~h(t)-g(t)\le h_0-g_0+{\epsilon },~h(t)\in [h_0, h_0+ \epsilon ],\; g(t)\in [g_0-\epsilon , g] \text{ for } t\in [0, s]. \end{aligned}$$

The rest of this section is devoted to proving the global existence and uniqueness of the solution to the problem (19). We will always assume that $\mathbf {(F),(F1), (F2)}$, $(\textbf{J})$, $(\textbf{K})$ hold, and the initial function pair $(u_{10}, u_{20})$ satisfies (14).

### Lemma 3.2

There exist positive constants $\epsilon _0, T_0, c_0$ such that

$$ \Gamma (\mathop \Sigma \limits _{c_0,s,\epsilon _0})\subseteq \mathop \Sigma \limits _{c_0,s,\epsilon _0} \text{ for } s\in (0, T_0]. $$

### Proof

Thanks to $\mathbf {(K)}$, there exists $\epsilon _0 \in (0, \frac{h_0}{4})$ such that $\int _{2\epsilon _0}^{3\epsilon _0}K_i(x) \textrm{d} x>0$, $i=1,2$. Define

$$\begin{aligned} T_0:=\frac{\epsilon _0}{4 K_0(\mu _1+\mu _2)(M_0^1+ M_0^2) (h_0-g_0+\epsilon _0)}>0, \end{aligned}$$

$$ c_0:=\sum _{i=1}^2\mu _i e^{-(d_i+L(M_0^1,M_0^2))T_0}\int _{2\epsilon _0}^{3\epsilon _0}K_i(x) \textrm{d} x \min _{x\in [g_0+\epsilon _0, h_0-\epsilon _0]}u_{i0}(x)>0, $$

where $$ L(M_0^1, M_0^2)$$ is the Lipschitz constant of $$f_i(t,x,\cdot ,\cdot )$$ over $$[0, M_0^1]\times [0, M_0^2]$$, and

24 $$\begin{aligned} K_0=\max \{\max \limits _{x\in [0, 2h_0-g_0]} K_1(x),~~\max \limits _{x\in [0, 2h_0-g_0]} K_2(x) \}. \end{aligned}$$

Fix $s\in (0, T_0]$ and $(g,h)\in \Sigma _{c_0,s,\epsilon _0}$, we next show $(\tilde{g}, \tilde{h})\in \Sigma _{c_0,s,\epsilon _0}$.

It follows from (22) that $(\tilde{g}, \tilde{h})\in C^1([0, s])\times C^1([0, s])$, $\tilde{g} (0)=g_0$, $\tilde{h}(0)=h_0$ and

25 $$\begin{aligned} \left\{ \begin{aligned}&\tilde{g}'(t)=-\sum _{i=1}^2\mu _i \int _{g(t)}^{h(t)} v^{g,h}_i(t,x) K_i(x-g(t)) \textrm{d} x, \;\;&t \in (0, s],\\&\tilde{h}'(t)=\sum _{i=1}^2\mu _i \int _{g(t)}^{h(t)}v^{g,h}_i(t,x) K_i(h(t)-x) \textrm{d} x, \;\;&t \in (0, s] . \end{aligned} \right. \end{aligned}$$

In view of (20)-(21) and Lemma 3.1, we can see that

$$\begin{aligned} f_i(t,x,v_1^{g,h},v_2^{g,h} )\ge -L(M_0^1,M_0^2 )v_i^{g,h} \end{aligned}$$

and

$$\begin{aligned} \left\{ \begin{aligned}&\partial _t v_i^{g,h}(t,x)\ge -d_iv_i^{g,h}(t,x)-L(M_0^1,M_0^2)v_i^{g,h}(t,x), &  0<t\le s,~x\in (g(t),h(t)),\\&\ v_i^{g,h}(0,x)=u_{i0}(x), &  x\in [g_0,h_0], \end{aligned} \right. \end{aligned}$$

which implies, for $x\in [g_0, h_0],\; t\in [0, s]$,

26 $$\begin{aligned} v_i^{g,h}(t,x)\ge e^{-(d_i+L(M_0^1,M_0^2))t}u_{i0}(x)\ge e^{-(d_i+L(M_0^1,M_0^2))T_0}u_{i0}(x). \end{aligned}$$

This, combined with (14), (23), (25) and (26), implies that for $t\in [0, s]$,

$$\begin{aligned} \tilde{h}'(t)&\ge \sum _{i=1}^2\mu _i e^{-(d_i+L(M_0^1,M_0^2))T_0} \int _{g_0}^{h_0}K_i(h(t)-x)u_{i0}(x)\textrm{d}x \\&\ge \sum _{i=1}^2\mu _i e^{-(d_i+L(M_0^1,M_0^2))T_0}\int _{h_0-3\epsilon _0} ^{h_0-\epsilon _0} K_i(h(t)-x)\textrm{d}x \min _{x\in [h_0-3\epsilon _0, h_0-\epsilon _0]}u_{i0}(x) \\&= \sum _{i=1}^2\mu _i e^{-(d_i+L(M_0^1,M_0^2))T_0}\int _{h(t)-h_0+\epsilon _0} ^{h(t)-h_0+3\epsilon _0} K_i(x)\textrm{d}x \min _{x\in [h_0-3\epsilon _0, h_0-\epsilon _0]}u_{i0}(x) \\&\ge \sum _{i=1}^2\mu _i e^{-(d_i+L(M_0^1,M_0^2))T_0}\int _{2\epsilon _0}^{3\epsilon _0}K_i(x) \textrm{d} x \min _{x\in [h_0-3\epsilon _0, h_0-\epsilon _0]}u_{i0}(x) \\&\ge \sum _{i=1}^2\mu _i e^{-(d_i+L(M_0^1,M_0^2))T_0}\int _{2\epsilon _0}^{3\epsilon _0}K_i(x) \textrm{d} x \min _{x\in [g_0+\epsilon _0, h_0-\epsilon _0]}u_{i0}(x)=c_{0}. \end{aligned}$$

Similarly, $$\tilde{g}'(t)\le -c_{0}$$ for $$t\in [0, s]$$.

Finally, by (23), (25) and Lemma 3.1,

$$\begin{aligned} [\tilde{h}(t)-\tilde{g}(t)]'= &  \sum _{i=1}^2\mu _i \int _{g(t)}^{h(t)}v^{g,h}_i(t,x) K_i(h(t)-x) \textrm{d} x\\ &  +\sum _{i=1}^2\mu _i \int _{g(t)}^{h(t)}v^{g,h}_i(t,x) K_i(x-g(t)) \textrm{d} x \\ \le &  (\mu _1+\mu _2)(M_0^1+ M_0^2) \sum _{i=1}^2 \int _{g(t)}^{h(t)}[ K_i(h(t)-x)+ K_i(x-g(t)) ] \textrm{d} x \\ \le &  2 (\mu _1+\mu _2)(M_0^1+ M_0^2) \int _{0}^{h(t)-g(t)}[K_1(y)+K_2(y)]\textrm{d} y \\ \le &  2 (\mu _1+\mu _2)(M_0^1+ M_0^2) {\max _{x\in [0, 2h_0-g_0]} [K_1(x)+K_2(x)] } [h(t)-g(t)] \\ \le &  4 K_0 (\mu _1+\mu _2)(M_0^1+ M_0^2)(h_0-g_0+\epsilon _0)~~~~ \text{ for } t\in [0, s]. \end{aligned}$$

Combining with the definition of $T_0$, we deduce, for $t\in [0, s]$

$$\begin{aligned} \tilde{h}(t)-\tilde{g}(t)\le h_0-g_0 +4 T_0 K_0 (\mu _1+\mu _2)(M_0^1+ M_0^2) (h_0-g_0+\epsilon _0)= h_0-g_0+\epsilon _0. \end{aligned}$$

This completes the proof.

$$\square $$

### Lemma 3.3

There exists $T_*\in (0, T_0]$ such that $\Gamma$ is a contraction mapping on $\Sigma _{c_0,s,\epsilon _0}$ for $s\in (0, T_*]$, where constants $\epsilon _0, T_0, c_0$ are defined in Lemma 3.2.

### Proof

For $(g_j,h_j)\in \Sigma _{c_0,s,\epsilon _0}$, $j=1,2$, we set

$$ v_i^{j}(t,x)=v_{i}^{h_j,g_j}(t,x),~~\ \left( \tilde{h}_j,\tilde{g}_j \right) =\Gamma \left( h_j,g_j\right) , ~~~ \Delta ^*_s=\Delta _s^{g_1,h_1}\cup \Delta _s^{g_2,h_2},~~i=1,2. $$

Then, by (23), we have

$$ \max \{h_2(t)-g_1(t),h_1(t)-g_1(t), h_1(t)-g_2(t),h_2(t)-g_2(t)\}\le h_0-g_0+2\epsilon _0< 2h_0-g_0. $$

Without loss of generality, we define $K_i(x)=K_i(-x)$ for $x<0$. It follows from (24) and $\mathbf {(K)}$ that

$$\begin{aligned}&{\left| {\tilde{h}_1(t)-\tilde{h}_2(t)}\right| } \\&\quad \le ~\sum _{i=1}^{2}\mu _i\int _0^t\left| \int _{g_1(\tau )}^{h_1(\tau )} K_i(h_1(\tau )-x)v_i^1(\tau ,x)\textrm{d} x-\int _{g_2(\tau )}^{h_2(\tau )} K_i(h_2(\tau )-x)v_i^2(\tau ,x) \textrm{d} x\right| {\textrm{d}}\tau \\&\quad \le ~\sum _{i=1}^{2}\mu _i\Big (\int _0^t\int _{g_1(\tau )}^{h_1(\tau )} K_i(h_1(\tau )-x) \left| v_i^1(\tau ,x)\textrm{d} x-v_i^2(\tau ,x)\right| {\textrm{d}} x{\textrm{d}}\tau \\&\quad \quad +\int _0^t \left| \int _{g_1(\tau )}^{g_2(\tau )} K_i(h_1(\tau )-x)v_i^2(\tau ,x)\textrm{d} x\right| +\left| \int _{h_2(\tau )}^{h_1(\tau )} K_i(h_1(\tau )-x)v_i^2(\tau ,x)\textrm{d} x\right| {\textrm{d}}\tau \\&\quad \quad +\int _0^t \int _{g_2(\tau )}^{h_2(\tau )} \left| K_i(h_1(\tau )-x)-K_i(h_2(\tau )-x)\right| v_i^2(\tau ,x) \textrm{d} x {\textrm{d}}\tau \Big ) \\&\quad \le ~ \sum _{i=1}^{2}\mu _i\Big ( sK_0(2h_0-g_0) \Vert v_i^1-v_i^2\Vert _{C(\overline{\Delta }_{s}^*)} + s M_0^i K_0 \Vert g_1-g_2\Vert _{C([0,s])} \\&\quad \quad +s M_0^i K_0 \Vert h_1-h_2\Vert _{C([0,s])} + s M_0^i (2h_0-g_0)\hat{L}_i({2h_0-g_0})\Vert h_1-h_2\Vert _{C([0,s])}\Big ) \\&\quad \le ~ C_0s\Big [\sum _{i=1}^{2}\Vert v_i^1-v_i^2\Vert _{C(\overline{\Delta }_{s}^*)} +\Vert h_1-h_2\Vert _{C([0,s])}+\Vert g_1-g_2\Vert _{C([0,s])}\Big ], \end{aligned}$$

where $C_0=C_0(\mu _1,\mu _2, K_0, g_0, h_0, M_0^1,M_0^2)$, and $\hat{L}_i(2h_0-g_0)$ is the Lipschitz constant of $K_i(\cdot )$ over $[0, 2h_0-g_0]$. Similarly,

$$\begin{aligned} \Big |\tilde{g}_1(t)-\tilde{g}_2(t)\Big |\le&\, \widetilde{C}_0s \Big [\sum _{i=1}^{2}\Vert v_i^1-v_i^2\Vert _{C(\overline{\Delta }_{s}^*)}+ \Vert h_1-h_2\Vert _{C([0,s])}\\&\qquad \quad +\Vert g_1-g_2\Vert _{C([0,s])}\Big ] \text{ for }~t\in [0, s]. \end{aligned}$$

Hence,

27 $$\begin{aligned} \begin{aligned}&~\Vert \tilde{h}_1-\tilde{h}_2\Vert _{C([0,s])}+\Vert \tilde{g}_1-\tilde{g}_2 \Vert _{C([0,s])}\\&\quad \le (C_0+\widetilde{C}_0)s\Big [\sum _{i=1}^{2}\Vert v_i^1-v_i^2\Vert _{C(\overline{\Delta }_{s}^*)}+\Vert h_1-h_2\Vert _{C([0,s])}+\Vert g_1-g_2\Vert _{C([0,s])}\Big ]. \end{aligned} \end{aligned}$$

As in the proof of the second step of Theorem 2.1 in Du et al. (2022), by making use of (27) and the basic assumptions that $f_1$ and $f_2$ are locally Lipschitz continuous in $(v_i^1, v_i^2)$ uniformly in (*t*, *x*), we know that, for any $0<s\le T_*:=T_*(C_0,\widetilde{C}_0, d_1,d_2, u_{10},u_{20}, \epsilon _0, J_1,J_2, f_1,f_2 )\le T_0$,

$$\begin{aligned} \Vert \tilde{h}_1-\tilde{h}_2\Vert _{C([0,s])}+\Vert \tilde{g}_1-\tilde{g}_2 \Vert _{C([0,s])}\le \frac{1}{2}\left[ \Vert h_1-h_2\Vert _{C([0,s])} +\Vert g_1-g_2\Vert _{C([0,s])}\right] . \end{aligned}$$

The proof is now completed. $\square$

Next, we prove the following result, which implies Theorem 1.1 since $f_i$ in (12) satisfy the general assumption $\mathbf {(F), (F1), (F2)}$ and the initial conditions $u_{10}$, $u_{20}$ in (14) can satisfy (8) by taking $g_0=-h_0$.

### Theorem 3.4

Assume $\mathbf {(F), (F1), (F2)}$, $\mathbf {(J)}$ and $\mathbf {(K)}$ hold. Then for any given $-g_0$, $h_0>0$ and $u_{10}$, $u_{20}$ satisfying (14), problem (19) has a unique solution $(u_1, u_2, g, h)$ defined for all $t>0$. Moreover, for any $T>0$, $g\in \mathbb G_{T},\; h\in \mathbb H_{T}$, $(u_1, u_2)\in \mathbb {X}_T$ and

$$ 0<u_1\le \max \{||u_{10}||_{\infty },k_0\},~~~~ 0<u_2\le \max \{||u_{20}||_{\infty },\theta (k_0)\}~~~~\textrm{in}\ \Delta _T, $$

where $k_0$, $\theta (k_0)$ are defined in the assumptions $(\textbf{F1})$ and $(\textbf{F2})$.

### Proof

Thanks to Lemmas 3.2 and 3.3, we obtain that $\Gamma$ has a unique fixed point $(g_*,h_*)$ in $\Sigma _{c_0,s,\epsilon _0}$ for $s\in (0, T_*]$ by using the contraction mapping theorem, where $c_0$, $T_*$, $\epsilon _0$ come from Lemmas 3.2 and 3.3. Therefore, it follows from (22) and Lemma 3.1 that (19) has a unique solution $(u_{1*}, u_{2*}, g_*,h_*)$ for $t\in [0, T_*]$. By using the extension arguments, we can show that the solution $(u_{1*}, u_{2*}, g_*,h_*)$ on $[0, T_*]$ can be extended to $(0,\infty )$. We omit the details here due to the proof is almost identical to the fourth step of Theorem 2.1 in Du et al. (2022). $\square$

### Comparison principle

#### Lemma 3.5

(Comparison principle) Assume $(\textbf{J})$, $(\textbf{K})$ hold, and (*B*, *M*, *g*, *h*) is the solution of (12). Let $\overline{g}\in \mathbb G_{ \overline{g}(0), T},\; \overline{h}\in \mathbb H_{\overline{h}(0), T}$ and $\overline{B}$, $\overline{B}_t$, $\overline{M}$, $\overline{M}_t\in C(\overline{\Delta }_T^{\overline{g},\overline{h} })$. If $(\overline{B}, \overline{M})$ satisfies

28 $$\begin{aligned} {\left\{ \begin{array}{ll} \displaystyle \overline{B}_t\ge d_1 \int _{\overline{g}(t)}^{\overline{h}(t)}J_1(x-y) \overline{B}(t,y)\textrm{d}y-d_1 \overline{B}+a_1(e_1-\overline{B})\overline{M}-b_1 \overline{B}, & (t,x)\in \Delta _T^{\overline{g}, \overline{h}},\\ \displaystyle \overline{M}_t\ge d_2 \int _{\overline{g}(t)}^{\overline{h}(t)}J_2(x-y) \overline{M}(t,y)\textrm{d}y-d_2\overline{M}+a_2(e_2- \overline{M}) \overline{B}-b_2 \overline{M} , & (t,x)\in \Delta _T^{\overline{g}, \overline{h}},\\ \overline{B}(t,\overline{g}(t)), \overline{B}(t,\overline{h}(t))\in [0,{e_1]},~~ \overline{M}(t,\overline{g}(t)), \overline{M}(t,\overline{h}(t))\in [0,{e_2}], & t>0,\\ \displaystyle \overline{g}'(t)\le -\mu _1 \int _{\overline{g}(t)}^{\overline{h}(t)}K_1(x-\overline{g}(t))\overline{B}(t,x)\textrm{d}x -\mu _2 \int _{\overline{g}(t)}^{\overline{h}(t)}K_2(x-\overline{g}(t))\overline{M}(t,x)\textrm{d}x, & t>0,\\ \displaystyle \overline{h}'(t)\ge \mu _1 \int _{\overline{g}(t)}^{\overline{h}(t)}K_1(\overline{h}(t)-x)\overline{B}(t,x)\textrm{d}x + \mu _2 \int _{\overline{g}(t)}^{\overline{h}(t)}K_2(\overline{h}(t)-x)\overline{M}(t,x)\textrm{d}x, & t >0,\\ {e_1\ge }\overline{B}(0,x)\ge u_{10}(x),~~ {e_2\ge }\overline{M}(0,x)\ge u_{20}(x),~~\overline{g}(0)\le -h_0, ~~ \overline{h}(0)\ge h_0, \  & x\in [-h_0,h_0]. \end{array}\right. } \end{aligned}$$

Then $$[g(t),h(t)]\subset [\overline{g}(t),\overline{h}(t)]$$ and

$$\begin{aligned} \ B(t,x)\le \overline{B}(t,x),~~~M(t,x)\le \overline{M}(t,x) \text{ for } t\in (0, T],\; x\in [g(t), h(t)]. \end{aligned}$$

#### Proof

For small $\epsilon >0$ and $i=1,2$, let $(B_\epsilon ,M_\epsilon ,g_\epsilon ,h_\epsilon )$ denote the unique solution of (12) with $h_0$ replaced by $h_0^\epsilon :=(1-\epsilon )h_0$, $\mu$ replaced by $\mu ^\epsilon _i: =(1-\epsilon )\mu _i$ and $u_{i0}$ replaced by $u_{i0}^\epsilon \in C([-h_0^\epsilon , h_0^\epsilon ])$ with

$$0<u_{i0}^\epsilon (x)\le u_{i0}(x) \text{ for } x\in (-h_0^\epsilon , h_0^\epsilon ),\ u_0^\epsilon (\pm h_0^\epsilon )=0,~~ \lim _{\epsilon \rightarrow 0} \Vert u_{i0}^\epsilon -u_{i0}\Vert _{C([-h_0^\epsilon , h_0^\epsilon ])}=0. $$

We claim that $\overline{g}(t)< g_\epsilon (t)$, $\overline{h}(t)> h_\epsilon (t)$ for $t\in (0, T]$. Otherwise, it follows from $\overline{g}(0)\le -h_0<-h_0^\epsilon$, $\overline{h}(0)\ge h_0> h_0^\epsilon$ that there exists $0<t_*\le T$ such that

$$ h_\epsilon (t)<\bar{h}(t),\ g_\epsilon (t) >\overline{g}(t)\ \text {for}\ t\in (0,t_*)\ \text {and} \ [h_\epsilon (t_*) -\overline{h}(t_*)][ g_\epsilon (t_*)- \overline{g}(t_*)]=0. $$

Without loss of generality, we may assume that $h_\epsilon (t_*) =\overline{h}(t_*) \text{ and } g_\epsilon (t_*)\ge \overline{g}(t_*).$ It follows that

29 $$\begin{aligned} h_\epsilon '(t_*) \ge \overline{h}'(t_*). \end{aligned}$$

On the other hand, thanks to (8), (28) and Lemma 2.1, we know

$$\overline{ B}(t,x)\in (0, e_1],~~\overline{ M}(t,x)\in (0, e_2] ~~~\text{ for }~~~~ 0<t\le T, ~~~\overline{g}(t)<x<\overline{h}(t).$$

This, together with Lemma 2.1 and the fact that

$$\begin{aligned} \displaystyle (\overline{B}-B_\epsilon )_t\ge &  d_1 \int _{g_\epsilon (t)}^{ h_\epsilon (t)}J_1(x-y) [\overline{B}-{B_\epsilon }]\textrm{d}y-d_1 (\overline{B}-B_\epsilon ) \\  &  +a_1(e_1-\overline{B})(\overline{M}-M_\epsilon )-(a_1M _\epsilon +b_1) (\overline{B}-B_\epsilon ), \\ \displaystyle (\overline{M}-M_\epsilon )_t\ge &  d_2 \int _{g_\epsilon (t)}^{ h_\epsilon (t)}J_2(x-y) [\overline{M}-{M}_\epsilon ]\textrm{d}y-d_2 (\overline{M}-M_\epsilon ) \\  &  +a_2(e_2-\overline{M})(\overline{B}-B_\epsilon ) -(a_2B_\epsilon +b_2) (\overline{M}-M_\epsilon ) \end{aligned}$$

for $$0<t\le t_*, ~g_\epsilon (t)<x<h_\epsilon (t)$$, shows

$$ \overline{B}(t,x)-B_\epsilon (t,x)>0, ~~~ \overline{M}(t,x)-M_\epsilon (t,x)>0~~~\text{ for }~~~~ 0<t\le t_*, ~g_\epsilon (t)<x<h_\epsilon (t).$$

Therefore,

$$\begin{aligned} &  {\overline{h}'(t_*)-h_\epsilon '(t_*)} \\ &  \quad \ge \mu _1 \int _{\overline{g}(t_*)}^{\overline{h}(t_*)}K_1(\overline{h}(t_*)-x)\overline{B}(t_*,x)\textrm{d}x + \mu _2 \int _{\overline{g}(t_*)}^{\overline{h}(t_*)}K_2(\overline{h}(t_*)-x)\overline{M}(t_*,x)\textrm{d}x \\ &  \quad \quad - \mu _1^\epsilon \int _{g_\epsilon (t_*)}^{h_\epsilon (t_*)} K_1(h_\epsilon (t_*)-x)B_\epsilon (t_*,x)\textrm{d}x -\mu _2^\epsilon \int _{g_\epsilon (t_*)}^{h_\epsilon (t_*)}K_2 (h_\epsilon (t_*)-x)M_\epsilon (t_*,x)\textrm{d}x \\ &  \quad>\mu _1^\epsilon \int _{ g_\epsilon (t_*)}^{ h_\epsilon (t_*)}K_1( h_\epsilon (t_*)-x)[\overline{B}(t_*,x)-B_\epsilon (t_*,x)]\textrm{d}x \\ &  \quad \quad + \mu _2^\epsilon \int _{g(t_*)}^{h_\epsilon (t_*)}K_2( h_\epsilon (t_*)-x)[\overline{M}(t_*,x)- M_\epsilon (t_*,x)]\textrm{d}x>0, \end{aligned}$$

which is a contradiction to (29). This proves our claim and $\overline{B}(t,x)>B_\epsilon (t,x)$, $\overline{M}(t,x)>M_\epsilon (t,x)$ for $t\in (0,T]$, $x\in [g_\epsilon (t), h_\epsilon (t)]$. Since the unique solution of (12) depends continuously on the parameters in (12), the desired result then follows by letting $\epsilon \rightarrow 0$. $\square$

To emphasize the dependence on the parameters $\mu _1$ and $\mu _2$, we denote the solution of problem (12) as $\left( B_{\mu _1,\mu _2}, M_{\mu _1,\mu _2}, g_{\mu _1,\mu _2}, h_{\mu _1,\mu _2}\right)$. The following result directly follows from Lemma 3.5.

#### Corollary 3.6

Suppose that **(J)** and **(K)** hold. If $\mu _1\le \mu _3$ and $\mu _2\le \mu _4$, then $h_{\mu _1,\mu _2} (t)\le h_{\mu _3,\mu _4} (t)$ and $g_{\mu _1,\mu _2} (t)\ge g_{\mu _3,\mu _4} (t)$ for $t>0$. Moreover, $B_{\mu _1,\mu _2}(t,x)\le B_{\mu _3,\mu _4}(t,x)$, $M_{\mu _1,\mu _2}(t,x)\le M_{\mu _3,\mu _4}(t,x)$ for all $t>0$ and $g_{\mu _1,\mu _2} (t)<x<h_{\mu _1,\mu _2} (t)$.

## Spreading-vanishing dichotomy and criteria

We will prove Theorems 1.2 and 1.3 in this section. Throughout this section, we always assume $(\textbf{J})$ and $(\textbf{K})$ hold, the initial function pair $(u_{10}, u_{20})$ satisfies (8), denote (*B*, *M*, *g*, *h*) as the unique positive solution of (12), and $g_\infty ,h_\infty$ are given by (13).

### Lemma 4.1

(i) If $h_{\infty }-g_{\infty }<\infty$, then $\lambda _1(g_\infty ,h_\infty )\ge 0$, where $\lambda _1(g_\infty , h_\infty )$ represents the principal eigenvalue obtained by substituting $[-L, L]$ with $[g_\infty , h_\infty ]$ in equation (16). Moreover,

30 $$\begin{aligned} \lim _{t\rightarrow \infty }||M||_{C[g(t),h(t)]}= \lim _{t\rightarrow \infty }||B||_{C[g(t),h(t)]}=0. \end{aligned}$$

(ii) If $h_{\infty }-g_{\infty }<\infty$ and $\mathcal R_0>1$, then $h_\infty -g_\infty \le 2L_*$, where $L_*$ comes from Proposition 2.4.

### Proof

(i) Firstly, we show $\lambda _1(g_\infty , h_\infty ) \ge 0$. Suppose, by contradiction, that $\lambda _1(g_\infty , h_\infty ) < 0$. By $(\textbf{K})$ and Proposition 2.4, there exist $\epsilon \in (0, \frac{h_0}{4})$ and $T \gg 1$ such that

31 $$\begin{aligned}&\int _{\epsilon }^{2h_0-2\epsilon } K_1(x) \textrm{d}x>0,~~h(t)>h_\infty -\epsilon ,~~\nonumber \\&\quad g(t)<g_\infty +\epsilon ,~~ \lambda _1(g(t),h(t))<0~~~\text{ for }~~~t\ge T. \end{aligned}$$

Let $(B_1(t,x),M_1(t,x))$ be the solution of (17) with $Q_L$ replaced by $(0,\infty )\times (g(T), h(T))$ and initial functions (*B*(*T*, *x*), *M*(*T*, *x*)). It follows from Lemma 2.1 and Remark 2.2 that

$$\begin{aligned} B_1(t,x)\le B(t+T,x), \ \ M_1(t,x)\le M(t+T,x) \ \ \ \ \textrm{for}\ (t,x)\in [0,\infty )\times [g(T),h(T)]. \end{aligned}$$

By Proposition 2.5-(iii), we deduce

$$\begin{aligned}&0<\widetilde{B}_1(x):=\lim _{t\rightarrow \infty } B_1(t,x)\le \liminf _{t\rightarrow \infty } B(t,x), \ \\&0<\widetilde{M}_1(x):=\lim _{t\rightarrow \infty } M_1(t,x)\le \liminf _{t\rightarrow \infty } M(t,x), \end{aligned}$$

hold uniformly on [*g*(*T*), *h*(*T*)]. Thus, there exists $T_1\ge T$ such that

$$\begin{aligned} 0< \frac{1}{2} \widetilde{B}_1(x)<B(t,x) \ \ \ \ \text{ for } t\ge T_1,\ x\in [g(T),h(T)]. \end{aligned}$$

This, together with (12) and (31), implies that for $t\ge T_1$,

$$\begin{aligned} \displaystyle h'(t)&= \mu _1 \int _{g(t)}^{h(t)}K_1(h(t)-x)B(t,x)\textrm{d}x +\mu _2 \int _{g(t)}^{h(t)}K_2(h(t)-x)M(t,x)\textrm{d}x \\ &\ge \mu _1 \int _{g_\infty +\epsilon }^{h_\infty -\epsilon }K_1(h(t)-x) \frac{1}{2} \widetilde{B}_1(x)\textrm{d}x \\ &\ge \frac{c_1\mu _1}{2}\int _{g_\infty +\epsilon }^{h_\infty -\epsilon }K_1(h(t)-x) \textrm{d}x = \frac{c_1\mu _1}{2}\int _ {h(t)-h_\infty +\epsilon }^{h(t)-g_\infty -\epsilon }K_1(x) \textrm{d}x \\ &\ge \frac{c_1\mu _1}{2}\int _{\epsilon }^{2h_0-2\epsilon } K_1(x) \textrm{d}x>0, \end{aligned}$$

where $c_1=\min \limits _{x\in [g(T), h(T)]} \widetilde{B}_1(x)>0.$ However, this contradicts to the fact $h_\infty <\infty$.

Let $(B_2(t,x),M_2(t,x))$ be the solution of (17) with $Q_L$ replaced by $(0,\infty )\times (g_\infty , h_\infty )$ through the initial functions $(e_1,e_2)$. It follows from Lemma 2.1 that

$$ 0\le B(t,x)\le B_2(t,x),\; 0\le M(t,x)\le M_2(t,x)\ \ \ \ \text{ for } t>0,\; x\in [g(t), h(t)]. $$

Since $\lambda _1(g_\infty , h_\infty )\ge 0$, by Proposition 2.5-(ii), we have $\lim \limits _{t\rightarrow \infty } (B_2, M_2)=(0,0)$ uniformly for $x\in [g_\infty , h_\infty ]$. Therefore $\lim \limits _{t\rightarrow \infty }||M||_{C[g(t),h(t)]}= \lim \limits _{t\rightarrow \infty }||B||_{C[g(t),h(t)]}=0.$

(ii) The conclusion then follows directly from $\lambda _1(g_\infty ,h_\infty )\ge 0$ and Proposition 2.4-(iii, iv). $\square$

### Lemma 4.2

If $\mathcal {R}_0< 1$ and $\limsup \limits _{y\rightarrow \infty }\frac{\int _0^y K_i(x)\textrm{d}x}{y}<\infty ~(i=1,2)$, then

$h_\infty -g_\infty <\infty$ and vanishing occurs.

### Proof

It follows from $\mathcal R_0=\sqrt{\frac{a_1a_2e_1e_2}{b_1b_2}}<1$ that $\frac{b_2}{a_2e_2}>\frac{a_1e_1}{b_1}$. Then there exists constant $0<\epsilon \ll 1$ such that $\frac{b_2-\epsilon }{a_2e_2}>\frac{a_1e_1}{b_1-\epsilon }$. Thus, there is $\delta :=\delta (\epsilon )>0$ such that

32 $$\begin{aligned} \frac{b_2-\epsilon }{a_2e_2}\ge \delta \ge \frac{a_1e_1}{b_1-\epsilon }. \end{aligned}$$

For $t\ge 0$ and $x\in {\mathbb R}$, define

$$\begin{aligned} \overline{B}(t,x):=\sigma \delta e^{-\epsilon t} , \ \ \overline{M}(t,x):=\sigma e^{-\epsilon t}, \end{aligned}$$

where

$$\begin{aligned} \sigma = \max \{ \max _{x\in [-h_0, h_0]}\frac{B(0,x)}{\delta }, ~~ \max _{x\in [-h_0, h_0]}M(0,x) \}. \end{aligned}$$

Let

$$\begin{aligned} \phi _1(t,x):=\overline{B}(t,x)- B(t,x),~~\phi _2(t,x):=\overline{M}(t,x)- M(t,x). \end{aligned}$$

Then for $t\ge 0$ and $- h_0\le x\le h_0$, we have

33 $$\begin{aligned} \phi _1(0,x)\ge 0, ~~\phi _2(0,x)\ge 0,~~ \phi _1(t,h(t))\ge 0, ~~\phi _2(t,h(t))\ge 0. \end{aligned}$$

Next, we claim that

34 $$\begin{aligned} \left\{ \begin{aligned} \partial _t\phi _1(t,x)\ge &  d_1\int _{g(t)}^{h(t)}J_1(x-y)\phi _1(t,y)\textrm{d}y-d_1\phi _1(t,x)-b_1\phi _1(t,x)+a_1e_1\phi _2(t,x), \\ \partial _t\phi _2(t,x)\ge &  d_2\int _{g(t)}^{h(t)}J_2(x-y)\phi _2(t,y)\textrm{d}y-d_2\phi _2(t,x)+a_2e_2\phi _1(t,x)-b_2\phi _2(t,x) \end{aligned} \right. \end{aligned}$$

for $(t,x)\in (0,\infty )\times (g(t),h(t))$. In fact, it follows from (12) and (32) that

$$\begin{aligned}&\partial _t\phi _1- d_1\int _{g(t)}^{h(t)}J_1(x-y)\phi _1(t,y)\textrm{d}y+d_1\phi _1+b_{1}\phi _1-a_1e_1\phi _2 \\&\quad = \overline{B}_t-B_t-d_1\int _{g(t)}^{h(t)}J_1(x-y)\overline{B}(t,y)\textrm{d}y+d_1\int _{g(t)}^{h(t)}J_1(x-y)B(t,y)\textrm{d}y \\&\quad \quad + d_1\overline{B}-d_1 B+b_1\overline{B}-b_1 B-a_1e_1\overline{M}+a_1e_1 M \\&\quad =\overline{B}_t -d_1\int _{g(t)}^{h(t)}J_1(x-y)B(t,y)\textrm{d}y+ d_1B -a_1(e_1-B)M+b_1 B\\&\qquad -d_1\int _{g(t)}^{h(t)}J_1(x-y)\overline{B}(t,y)\textrm{d}y+d_1\int _{g(t)}^{h(t)}J_1(x-y)B(t,y)\textrm{d}y+ d_1\overline{B}\\&\qquad -d_1 B+b_1\overline{B}-b_1 B-a_1e_1\overline{M}+a_1e_1 M \\ &\quad =-\epsilon \overline{B}-d_1\int _{g(t)}^{h(t)}J_1(x-y)\overline{B}(t,y)\textrm{d}y+d_1\overline{B}+b_1\overline{B}-a_1e_1 \overline{M}+a_1BM \\&\quad \ge (b_1-\epsilon )\overline{B}-a_1e_1 \overline{M} \\&\quad = (b_1-\epsilon )\sigma \delta e^{-\epsilon t} -a_1e_1 \sigma e^{-\epsilon t}=[\delta (b_1-\epsilon ) -a_1e_1]\sigma e^{-\epsilon t}\ge 0. \end{aligned}$$

Similarly,

$$\begin{aligned}&\partial _t\phi _2- d_2\int _{g(t)}^{h(t)}J_2(x-y)\phi _2(t,y)\textrm{d}y+d_2\phi _2-a_2e_2\phi _1+b_{2}\phi _2 \\&\quad = \overline{M}_t-M_t-d_2\int _{g(t)}^{h(t)}J_2(x-y)\overline{M}(t,y)\textrm{d}y+d_2\int _{g(t)}^{h(t)}J_2(x-y)M(t,y)\textrm{d}y \\&\quad \quad +d_2\overline{M}-d_2 M-a_2e_2\overline{B}+a_2e_2B+b_{2}\overline{M}-b_{2} M \\&\quad =-\epsilon \overline{M}-d_2\int _{g(t)}^{h(t)}J_2(x-y)\overline{M}(t,y)\textrm{d}y+d_2\overline{M}+b_2\overline{M}-a_2e_2 \overline{B}+a_2BM \\&\quad \ge (b_2-\epsilon )\overline{M}-a_2e_2 \overline{B} \\&\quad = (b_2-\epsilon )\sigma e^{-\epsilon t} -a_2e_2 \sigma \delta e^{-\epsilon t}=[ (b_2-\epsilon ) -a_2e_2\delta ]\sigma e^{-\epsilon t}\ge 0. \end{aligned}$$

Hence, by (33)–(34) and Lemma 2.1, we obtain

$$\begin{aligned} B(t,x)\le \sigma \delta e^{-\epsilon t} , ~~ M(t,x)\le \sigma e^{-\epsilon t} ~~~\text{ for }~~~(t,x)\in (0,\infty )\times (g(t),h(t)). \end{aligned}$$

This, together with $\limsup \limits _{y\rightarrow \infty }\frac{\int _0^y K_i(x)\textrm{d}x}{y}<\infty ~(i=1,2)$, indicates there exists $c_0>0$ such that

$$\begin{aligned} \displaystyle h'(t)&= \mu _1 \int _{g(t)}^{h(t)}K_1(h(t)-x)B(t,x)\textrm{d}x + \mu _2 \int _{g(t)}^{h(t)}K_2(h(t)-x)M(t,x)\textrm{d}x \\ &\le \mu _1 \sigma \delta e^{-\epsilon t}\int _{g(t)}^{h(t)}K_1(h(t)-x)\textrm{d}x+ \mu _2 \sigma e^{-\epsilon t}\int _{g(t)}^{h(t)}K_2(h(t)-x)\textrm{d}x \\ &=\mu _1 \sigma \delta e^{-\epsilon t}\int _{0}^{h(t)-g(t)}K_1(x)\textrm{d}x+ \mu _2 \sigma e^{-\epsilon t}\int _{0}^{h(t)-g(t)}K_2(x)\textrm{d}x \\ &\le \mu _1\sigma \delta e^{-\epsilon t}c_0[h(t)-g(t)]+ \mu _2 \sigma e^{-\epsilon t}c_0[h(t)-g(t)] \\ &=(\mu _1 \delta + \mu _2 ) \sigma c_0 e^{-\epsilon t}[h(t)-g(t)] :=C e^{-\epsilon t}[h(t)-g(t)]~~~~\text{ for }~~t\ge 0. \end{aligned}$$

Similarly

$$\begin{aligned} - \displaystyle g'(t)\le (\mu _1 \delta + \mu _2 ) \sigma c_0 e^{-\epsilon t}[h(t)-g(t)] =C e^{-\epsilon t}[h(t)-g(t)]~~~~\text{ for }~~t\ge 0. \end{aligned}$$

Therefore

$$\begin{aligned} (h(t)-g(t))'\le 2Ce^{-\epsilon t}(h(t)-g(t))~~~\text{ for }~~~t\ge 0, \end{aligned}$$

which implies $$h_\infty -g_\infty <\infty .$$ $$\square $$

### Lemma 4.3

If $\mathcal {R}_0= 1$ and **(K1)** holds, then $h_\infty -g_\infty <\infty$ and vanishing occurs.

### Proof

We only need to show $h_\infty -g_\infty <\infty$ due to Lemma 4.1. It follows from **(J)** and **(K1)** that

$$\begin{aligned}&\int _{g(t)}^{h(t)}\left[ \int _{g(t)}^{h(t)} J_1(x-y)B(t,y)\textrm{d}y-B(t,x) \right] \textrm{d}x\\&\quad =\int _{g(t)}^{h(t)}\int _{g(t)}^{h(t)} J_1(x-y)[B(t,y)-B(t,x)]\textrm{d}y\textrm{d}x\\&\quad \quad -\int _{g(t)}^{h(t)}\int _{h(t)}^{\infty }J_1(x-y) B(t,x)\textrm{d}y\textrm{d}x-\int _{g(t)}^{h(t)}\int _{-\infty }^{g(t)}J_1(x-y) B(t,x)\textrm{d}y\textrm{d}x\\&\quad =-\int _{g(t)}^{h(t)}B(t,x) \left[ \int _{h(t)-x}^{\infty }J_1(y) \textrm{d}y +\int _{x-g(t)}^{\infty }J_1(y) \textrm{d}y \right] \textrm{d}x \\&\quad \le - \kappa _1 \int _{g(t)}^{h(t)}B(t,x)\Big [ K_1(h(t)-x) +K_1( x-g(t))\Big ]\textrm{d}x. \end{aligned}$$

Similarly,

$$\begin{aligned}&\int _{g(t)}^{h(t)}\left[ \int _{g(t)}^{h(t)} J_2(x-y)M(t,y)\textrm{d}y-M(t,x) \right] \textrm{d}x \\&\quad =-\int _{g(t)}^{h(t)}\int _{h(t)}^{\infty }J_2(x-y) M(t,x)\textrm{d}y\textrm{d}x-\int _{g(t)}^{h(t)}\int _{-\infty }^{g(t)}J_2(x-y) M(t,x)\textrm{d}y\textrm{d}x \\&\quad =-\int _{g(t)}^{h(t)}M(t,x)\Big [ \int _{h(t)-x}^{\infty }J_2(y) \textrm{d}y +\int _{x-g(t)}^{\infty }J_2(y) \textrm{d}y \Big ]\textrm{d}x \\ &\quad \le - \kappa _2 \int _{g(t)}^{h(t)}M(t,x)\Big [ K_2(h(t)-x) +K_2( x-g(t))\Big ]\textrm{d}x. \end{aligned}$$

Thus,

$$\begin{aligned}&\int _{g(t)}^{h(t)}d_1\left[ \int _{g(t)}^{h(t)} J_1(x-y)B(t,y)\textrm{d}y-B(t,x) \right] \\&\quad \quad +\frac{a_1e_1 d_2}{b_2} \left[ \int _{g(t)}^{h(t)} J_2(x-y)M(t,y)\textrm{d}y-M(t,x) \right] \textrm{d}x \\&\quad \le - d_1\kappa _1 \int _{g(t)}^{h(t)}B(t,x)\Big [ K_1(h(t)-x) +K_1( x-g(t))\Big ]\textrm{d}x \\&\quad \quad - \frac{a_1e_1\kappa _2 d_2}{b_2} \int _{g(t)}^{h(t)}M(t,x)\Big [ K_2(h(t)-x) +K_2( x-g(t))\Big ]\textrm{d}x \\&\quad =-\frac{ d_1\kappa _1}{\mu _1} \mu _1 \int _{g(t)}^{h(t)}B(t,x) K_1(h(t)-x) \textrm{d}x- \frac{a_1e_1\kappa _2 d_2}{b_2\mu _2} \mu _2 \int _{g(t)}^{h(t)}M(t,x) K_2(h(t)-x)\textrm{d}x \\ &\quad \quad - \frac{ d_1\kappa _1}{\mu _1} \mu _1 \int _{g(t)}^{h(t)}B(t,x) K_1(x-g(t)) \textrm{d}x- \frac{a_1e_1\kappa _2 d_2}{b_2\mu _2} \mu _2 \int _{g(t)}^{h(t)}M(t,x) K_2(x-g(t))\textrm{d}x \\ &\quad \le \sigma \Big [- \mu _1 \int _{g(t)}^{h(t)}B(t,x) K_1(h(t)-x) \textrm{d}x- \mu _2 \int _{g(t)}^{h(t)}M(t,x) K_2(h(t)-x)\textrm{d}x \Big ] \\ &\qquad +\sigma \Big [- \mu _1 \int _{g(t)}^{h(t)}B(t,x) K_1(x-g(t)) \textrm{d}x- \mu _2 \int _{g(t)}^{h(t)}M(t,x) K_2(x-g(t))\textrm{d}x \Big ] \\ &\quad = -\sigma [h'(t)-g'(t)], \end{aligned}$$

where $\sigma :=\min \{ \frac{ d_1\kappa _1}{\mu _1}, ~~\frac{a_1e_1\kappa _2 d_2}{b_2\mu _2}\}>0$. Therefore, by making use of (12) and $\mathcal R_0= 1$, we obtain

$$\begin{aligned}&\frac{\textrm{d}}{\textrm{d}t}\int _{g(t)}^{h(t)} \left[ B(t,x)+\frac{a_1e_1}{b_2}M(t,x)\right] \textrm{d}x\\ =&\int _{g(t)}^{h(t)} \left[ B_t(t,x)+\frac{a_1e_1}{b_2}M_t(t,x)\right] \textrm{d}x \\ =&\int _{g(t)}^{h(t)} d_1\left[ \int _{g(t)}^{h(t)} J_1(x-y)B(t,y)\textrm{d}y-B(t,x) \right] +a_1(e_1-B)M-b_1 B \\&+\frac{a_1e_1}{b_2}\Big \{d_2\left[ \int _{g(t)}^{h(t)} J_2(x-y)M(t,y)\textrm{d}y-M(t,x) \right] +a_2(e_2-M)B-b_2 M\Big \} \textrm{d}x \\ \le&\;- \sigma [h'(t)-g'(t)]+\int _{g(t)}^{h(t)} \left[ a_1(e_1-B)M-b_1 B +\frac{a_1e_1}{b_2}\Big (a_2(e_2-M)B-b_2 M\Big ) \right] \textrm{d}x\\ =&\;-\sigma [h'(t)-g'(t)]+\int _{g(t)}^{h(t)}\left[ b_1(\mathcal R_0-1)B-\big (\frac{a_1a_2e_1}{b_2}+a_1\big )HV\right] \textrm{d}x\\ \le&\;-\sigma [h'(t)-g'(t)]. \end{aligned}$$

As a result, for all $t> 0$ we have

$$\begin{aligned} \sigma [h(t)-g(t)]\le&\int _{g(t)}^{h(t)} \left( B(t,x)+\frac{a_1e_1}{b_2}M(t,x)\right) \textrm{d}x+\sigma [h(t)-g(t)]\\ \le&\int _{g(0)}^{h(0)} \left( B(0,x)+\frac{a_1e_1}{b_2}M(0,x)\right) \textrm{d}x+\sigma [h(0)-g(0)], \end{aligned}$$

which clearly implies $h_{\infty }-g_{\infty }<\infty$. $\square$

### Remark 4.4

It is not difficult to find that the proof of Lemma 4.3 is also valid for the case $\mathcal R_0<1$. Moreover, if $K_i$ ($i=1,2$) satisfy condition ***(K1)***, then by ***(J)*** it follows that $K_i\in L^\infty ([0,\infty ))$, which implies $\limsup \limits _{y\rightarrow \infty }\frac{\int _0^y K_i(x)\textrm{d}x}{y}<\infty ~(i=1,2)$. Therefore, if $\mathcal {R}_0\le 1$ and **(K1)** holds, then $h_\infty -g_\infty <\infty$ and vanishing occurs.

### Remark 4.5

To address the difficulty arising from the independence of the boundary kernel function $K_i$ and the dispersal kernel function $J_i$, we adopt some new ideas and techniques in our analysis. In particular, under the assumption $\mathcal R_0<1$ (see Lemma 4.2), a new pair of upper solutions is constructed, which differs substantially from the approaches used in Lin and Zhu (2017) and Du and Ni (2020). For the critical case $\mathcal {R}_0 = 1$, condition **(K1)** is imposed in Lemma 4.3, revealing a subtle interplay between the dispersal condition and the free boundary condition.

### Lemma 4.6

Let $\lambda _1(-h_0,h_0)$ be the principal eigenvalue of (16) with $L=h_0$. If $\lambda _1(-h_0,h_0)>0$ and $||u_{10}||_{C[-h_0,h_0]}+||u_{20}||_{C[-h_0,h_0]}$ is sufficiently small, then

$$\begin{aligned} \lim _{t\rightarrow \infty }||M||_{C[g(t),h(t)]}=\lim _{t\rightarrow \infty }||B||_{C[g(t),h(t)]}=0. \end{aligned}$$

### Proof

To prove this result, according to Lemma 4.1, we only need to show $h_\infty -g_\infty <\infty$. Note that $\lambda _1(-h_0,h_0)>0$ and $\lambda _1(-L,L)$ is continuous for $L\in (0,\infty )$ by Proposition 2.4-(i), then there exists constant $h_1>h_0$ such that $\lambda _1(-h_1, h_1)>0$. Let $(\phi ,\psi )$ be a positive eigenfunction pair corresponding to $\lambda _1(-h_1, h_1)$.

Now, for $t\in [0,\infty )$, $x\in [-h_1, h_1]$, we define

$$\begin{aligned}&\overline{h}(t):=h_0+ (h_1-h_0)[1-e^{-\delta t}],\ \overline{g}(t):=-\overline{h}(t), \ \ \\&\overline{B}(t,x):=ce^{-\delta t} \phi (x), \ \ \overline{M}(t,x):=ce^{-\delta t} \psi (x), \end{aligned}$$

where $$\delta ={\lambda _1(-h_1, h_1)},$$

$$\begin{aligned} c=&\min \Big \{\frac{e_1}{\max \limits _{|x|\le h_1}\phi (x)}, ~~\frac{e_2}{\max \limits _{|x|\le h_1}\psi (x)}, \\&\quad \quad ~~ \frac{ \delta (h_1-h_0)}{\mu _1 \max \limits _{x\in [0,2h_1]}K_1(x)\int _{-h_1}^{h_1}\phi (x)\textrm{d}x+ \mu _2 \max \limits _{x\in [0,2h_1]}K_2(x)\int _{-h_1}^{h_1}\psi (x)\textrm{d}x }\Big \}. \end{aligned}$$

Then, for $t\in [0,\infty )$ and $x\in [-h_0,h_0]$, we obtain $\overline{h}(t)\in [h_0, h_1)$,

$$\begin{aligned} \overline{B}(0,x)=c\phi (x)\le c\max \limits _{|x|\le h_1}\phi (x)\le e_1,\ \ \ \overline{M}(0,x)=c\psi (x)\le c\max \limits _{|x|\le h_1}\psi (x)\le e_2, \end{aligned}$$

and

$$\begin{aligned}&0\le \overline{B}(t,\overline{g}(t))=ce^{-\delta t} \phi (-\overline{h}(t))\le c\max \limits _{|x|\le h_1}\phi (x)\le e_1,\ \\&0\le \overline{B}(t,\overline{h}(t))\le c\max \limits _{|x|\le h_1}\phi (x)\le e_1,\\&0\le \overline{M}(t,\overline{g}(t))=ce^{-\delta t} \psi (-\overline{h}(t))\le c\max \limits _{|x|\le h_1}\psi (x)\le e_2,\ \\&0\le \overline{M}(t,\overline{h}(t))\le c\max \limits _{|x|\le h_1}\psi (x)\le e_2. \end{aligned}$$

Next, we will show that $(\overline{M},\overline{B},\overline{g},\overline{h})$ is an upper solution of (12). Thanks to (16), we deduce

$$\begin{aligned}&\displaystyle \overline{B}_t-d_1 \int _{\overline{g}(t)}^{\overline{h}(t)}J_1(x-y)\overline{B}(t,y)\textrm{d}y+d_1\overline{B}(t,x)-a_1(e_1 -\overline{B})\overline{M}+b_1 \overline{B}\\&\quad \ge \displaystyle \overline{B}_t-d_1 \int _{-h_1}^{h_1}J_1(x-y)\overline{B}(t,y)\textrm{d}y+d_1\overline{B}(t,x)-a_1e_1\overline{M}+b_1 \overline{B}\\&\quad =-\delta \overline{B}+\lambda _1(-h_1,h_1)\overline{B}= 0\ \ \ \ \ \ \ \textrm{for}\ t>0, \; x\in (\overline{g}(t),\overline{h}(t)). \end{aligned}$$

Similarly,

$$\begin{aligned}&\displaystyle \overline{M}_t- d_2 \int _{\overline{g}(t)}^{\overline{h}(t)}J_2(x-y)\overline{M}(t,y)\textrm{d}y-d_2\overline{M}(t,x)-a_2(e_2-\overline{M})\overline{B}+b_2\overline{M} \ge 0. \end{aligned}$$

Note that $[\overline{g}(t),\overline{h}(t)]\subset (-h_1,h_1)$ and $\overline{h}(t)-\overline{g}(t)\le 2h_1$ for all $t\ge 0$, we have

$$\begin{aligned}&\mu _1 \int _{\overline{g}(t)}^{\overline{h}(t)}K_1(\overline{h}(t)-x)\overline{B}(t,x)\textrm{d}x + \mu _2 \int _{\overline{g}(t)}^{\overline{h}(t)}K_2(\overline{h}(t)-x)\overline{M}(t,x)\textrm{d}x \\&\quad \le \mu _1 \max \limits _{x\in [0,2h_1]}K_1(x)\int _{\overline{g}(t)}^{\overline{h}(t)} \overline{B}(t,x)\textrm{d}x+ \mu _2 \max \limits _{x\in [0,2h_1]}K_2(x) \int _{\overline{g}(t)}^{\overline{h}(t)} \overline{M}(t,x)\textrm{d}x \\&\quad \le \mu _1 \max \limits _{x\in [0,2h_1]}K_1(x)\int _{-h_1}^{h_1} ce^{-\delta t} \phi (x) \textrm{d}x+ \mu _2 \max \limits _{x\in [0,2h_1]}K_2(x) \int _{-h_1}^{h_1} ce^{-\delta t} \psi (x)\textrm{d}x \\&\quad \le \delta (h_1-h_0)e^{-\delta t} =\overline{h}'(t),\ \ \ t> 0, \end{aligned}$$

and similarly,

$$\begin{aligned} -\mu _1 \int _{\overline{g}(t)}^{\overline{h}(t)}K_1(x-\overline{g}(t))\overline{B}(t,x)\textrm{d}x - \mu _2 \int _{\overline{g}(t)}^{\overline{h}(t)}K_2(x-\overline{g}(t))\overline{M}(t,x)\textrm{d}x \ge \overline{g}'(t), \ \ \ t> 0. \end{aligned}$$

Clearly we also have

$$\begin{aligned} u_{10}(x)\le c\phi (x)= \overline{B}(0,x),\ \ \ u_{20}(x)\le c\psi (x)=\overline{M}(0,x) \ \ \textrm{for}\ x\in [-h_0,h_0], \end{aligned}$$

provided that

$$ ||u_{10}||_{C[-h_0,h_0]}\le c\min _{x\in [-h_0, h_0]}\phi (x), ~~~~ ||u_{20}||_{C[-h_0,h_0]}\le c\min _{x\in [-h_0,h_0]}\psi (x). $$

We are now ready to apply Lemma 3.5 to conclude that

$$ {[}g(t), h(t)]\subset [\overline{g}(t), \overline{h}(t)] \text{ and } \text{ hence } h_\infty -g_\infty \le \overline{h}(\infty )-\overline{g}(\infty )=2h_1. $$

The proof is now complete. $\square$

### Remark 4.7

From the definition of *c* in Lemma 4.6, it follows that for given initial function pair $(u_{10}, u_{20})$ satisfying (8), there exists $\underline{\mu }:=\underline{\mu }(u_{10}, u_{20})>0$ such that vanishing happens for (12) when $\mu _1+\mu _2\in (0, \underline{\mu }]$ and $\lambda _1(-h_0,h_0)>0$.

### Lemma 4.8

If $\lambda _1(g(t_0), h(t_0))<0$ for some $t_0\ge 0$, then $-g_\infty =h_\infty =+\infty$. Further assuming that **(K1)** holds or $\mathcal { R}_0>1$, we can obtain

35 $$\begin{aligned} \lim _{t\rightarrow \infty }M(t,x)=M^*,\ \ \lim _{t\rightarrow \infty }B(t,x)=B^*\ \ \ \ \mathrm{locally\ uniformly\ in}\ {\mathbb R}, \end{aligned}$$

where $\lambda _1(g(t_0), h(t_0))$ is the eigenvalue of (16) with $[-L,L]$ replaced by $[g(t_0), h(t_0)]$.

### Proof

We first claim that $h_\infty -g_\infty =\infty$. Suppose, on the contrary, $h_\infty -g_\infty <\infty$. Then, by Lemma 4.1, we deduce

$$ \lambda _1(g_\infty ,h_\infty )\ge 0, $$

which together with Proposition 2.4-(i) yields that

$$\begin{aligned} \lambda _1(g(t),h(t))\ge 0 \ \ \ \textrm{for}\ t\ge 0. \end{aligned}$$

However, this leads to a contradiction to $\lambda _1(g(t_0), h(t_0))<0$ for some $t_0\ge 0$.

Next, we prove that $-g_\infty =h_\infty =+\infty$. Without loss of generality, we assume that $h_\infty <+\infty$ and $g_\infty =-\infty$. Hence, as in the proof of Lemma 4.1, by making use of $h_\infty <+\infty$ and $\lambda _1(g(t_0), h(t_0))<0$ for some $t_0\ge 0$, we can find some constants $\delta >0$ and $T_1>t_0$ such that

$$\begin{aligned} \displaystyle h'(t)&= \mu _1 \int _{g(t)}^{h(t)}K_1(h(t)-x)B(t,x)\textrm{d}x +\mu _2 \int _{g(t)}^{h(t)}K_2(h(t)-x)M(t,x)\textrm{d}x\ge \delta >0\\ \end{aligned}$$

for $t>T_1$. However, this leads to a contradiction to the fact $h_\infty <\infty$.

Finally, we show (35) holds. Let $(\overline{B}(t),\overline{M}(t))$ be the unique positive solution of the following ordinary differential equations:

$$\begin{aligned} {\left\{ \begin{array}{ll} \displaystyle \overline{B}_t= a_1(e_1- \overline{B})\overline{M}-b_1 \overline{B}, \ \ \ \  & t>0,\\ \displaystyle \overline{M}_t= a_2(e_2-\overline{M})\overline{B}-b_2\overline{M}, \ \ \ \  & t>0,\\ \overline{B}(0)= e_1,\ \overline{M}(0)= e_2. \end{array}\right. } \end{aligned}$$

Then, by Lemma 2.1, we have

$$\begin{aligned} M(t,x)\le \overline{M}(t),\ \ \ B(t,x)\le \overline{B}(t)\ \ \ \ \ \textrm{for}\ t>0, \; x\in [g(t),h(t)]. \end{aligned}$$

Note that $h_\infty -g_\infty =\infty$ and **(K1)** holds. Based on Remark 4.4, we obtain that $\mathcal { R}_0>1$. Hence,

$$\begin{aligned} \lim _{t\rightarrow \infty }\overline{B}(t)=B^*,\ \ \ \lim _{t\rightarrow \infty }\overline{M}(t)=M^*, \end{aligned}$$

which implies

36 $$\begin{aligned} B^*\ge \limsup _{t\rightarrow \infty } B(t,x),\ \ M^* \ge \limsup _{t\rightarrow \infty } M(t,x)&\;\;\; \mathrm{locally\ uniformly\ in}\ {\mathbb R}. \end{aligned}$$

On the other hand, for fixed $s\ge t_0$, we define $(B_s(t,x),M_s(t,x))$ to be a solution of (17) with $Q_L$ replaced by $(0,\infty )\times [g(s), h(s)]$, and initial functions $B_s(0,x)=B(s,x)$ and $M_s(0,x)=M(s,x)$. Noting that $\lambda _1(g(s),h(s))<0$, by applying Lemma 2.1 and Proposition 2.5-(iii), we have

$$\begin{aligned}&0<\widetilde{M}_s(x):=\lim _{t\rightarrow \infty } M_s(t,x)\le \liminf _{t\rightarrow \infty } M(t+s,x) &  \mathrm{uniformly\ in}\ [g(s),h(s)],\\&0<\widetilde{B}_s(x):=\lim _{t\rightarrow \infty } B_s(t,x)\le \liminf _{t\rightarrow \infty } B(t+s,x) &  \mathrm{uniformly\ in}\ [g(s),h(s)], \end{aligned}$$

where $(\widetilde{M}_s(x),\widetilde{B}_s(x))$ is the positive solution of (18) with $[-L, L]$ replaced by [*g*(*s*), *h*(*s*)]. This, combined with Proposition 2.5 and $(g(s), h(s))\rightarrow (-\infty ,\infty )$ as $s\rightarrow \infty$, shows that

$$\begin{aligned}&B^* \le \liminf _{t\rightarrow \infty } B(t+s,x)=\liminf _{t\rightarrow \infty } B(t,x) &  \mathrm{locally\ uniformly\ in}\ {\mathbb R},\\&M^*\le \liminf _{t\rightarrow \infty } M(t+s,x)=\liminf _{t\rightarrow \infty } M(t,x) &  \mathrm{locally\ uniformly\ in}\ {\mathbb R}, \end{aligned}$$

which together with (36) proves (35). $\square$

Now we are ready to present the proofs of two main theorems.

### Proof of Theorem 1.2

If $h_\infty -g_\infty <\infty$, then (30) holds by Lemma 4.1-(i). If $h_\infty -g_\infty =\infty$, then by Remark 4.4, we must have $\mathcal { R}_0>1$. These, combined with Proposition 2.4-(iii, iv), imply that there exists $t_0\gg 1$ such that $\lambda _1(g(t_0),h(t_0))<0$. Therefore, $-g_\infty =h_\infty =\infty$ and (35) holds by Lemma 4.8. $\square$

### Proof of Theorem 1.3

We provide arguments for each item individually:

(i) It follows directly from Lemma 4.2.
(ii) This item follows directly from Lemma 4.3.
(iii) Thanks to Proposition 2.4-(iii, iv), we obtain $\lambda _1(-h_0,h_0)<0$. Then spreading happens by Lemma 4.8.
(iv) In view of Proposition 2.4-(iv), we know that $\lambda _1(-h_0,h_0)>0$.
  - The statement holds due to Lemma 4.6.
  - Similar to Corollary (), we use $(B_{\mu _1,\mu _2}, M_{\mu _1,\mu _2}, g_{\mu _1,\mu _2}, h_{\mu _1,\mu _2})$ to stress the dependence of the unique positive solution (*B*, *M*, *g*, *h*) of (12) on $\mu _1$ and $\mu _2$. Based on Remark 4.7, there exists constant $\underline{\mu }>0$ such that vanishing happens for $\mu _1+\mu _2\in (0, \underline{\mu }]$. Next, we claim that there exists constant $\overline{\mu }>0$ such that spreading happens for $\mu _1+\mu _2 \in (\overline{\mu }, +\infty )$. Otherwise, for any $\mu _1+\mu _2\in (0,\infty )$, we have
    $$\begin{aligned} h_{\mu _1,\mu _2}^{\infty }-g_{\mu _1,\mu _2}^{\infty }:=\lim _{t\rightarrow +\infty } h_{\mu _1,\mu _2}(t)- \lim _{t\rightarrow +\infty } g_{\mu _1,\mu _2}(t) <+\infty . \end{aligned}$$
    By Lemma 4.1-(ii) and $\mathcal R_0>1$, we have $h_{\mu _1,\mu _2}^{\infty }-g_{\mu _1,\mu _2}^{\infty }\le 2L_*$. This, combined with Corollary 3.6, implies
    $$\begin{aligned} H_{\infty }- G_{\infty }:=\lim _{\mu _1,\mu _2 \rightarrow +\infty }h_{\mu _1,\mu _2}^{\infty }- \lim _{\mu _1,\mu _2 \rightarrow +\infty }g_{\mu _1,\mu _2}^{\infty }\le 2L_*. \end{aligned}$$
    In view of **(K)**, there exists $\varepsilon _0\in (0, \frac{h_0}{4})$ such that
    $$\begin{aligned} \delta _0:=\min \Big \{ \int _{2\varepsilon _0}^{ 2h_0-\varepsilon _0} K_1(x)\textrm{d}x,~~\int _{2\varepsilon _0}^{ 2h_0-\varepsilon _0} K_2(x)\textrm{d}x\Big \} >0. \end{aligned}$$
    For such $\varepsilon _0$, there exist $\mu _0, t_0>0$ such that
    $$0\le H_{\infty }-h_{\mu _1,\mu _2}^{\infty }\le \frac{\varepsilon _0}{2},\ \ 0\le h_{\mu _1,\mu _2}^{\infty }-h_{\mu _1,\mu _2}(t)\le \frac{\varepsilon _0}{2}~\text{ for } ~\mu _1,\mu _2\ge \mu _0,\ \ t\ge t_0,$$
    and thus $H_{\infty }- h_{\mu _0,\mu _0}(t)\le \varepsilon _0$ for $t\ge t_0$. As a result, for $\mu _1,\mu _2\ge \mu _0$ and $t\ge t_0$,
    $$\begin{aligned} &  h_{\mu _1,\mu _2}'(t) \\= &  \mu _1 \int _{g_{\mu _1,\mu _2}(t)}^{h_{\mu _1,\mu _2}(t)} K_1(h_{\mu _1,\mu _2}(t)-x)B_{\mu _1,\mu _2}(t,x)\textrm{d}x \\  &  + \mu _2\int _{g_{\mu _1,\mu _2}(t)}^{h_{\mu _1,\mu _2}(t)} K_2(h_{\mu _1,\mu _2}(t)-x)M_{\mu _1,\mu _2}(t,x)\textrm{d}x \\ \ge &  \mu _1 \int _{g_{\mu _0,\mu _0}(t)}^{h_{\mu _0,\mu _0}(t)} K_1(h_{\mu _1,\mu _2}(t)-x)B_{\mu _0,\mu _0}(t,x)\textrm{d}x \\  &  + \mu _2\int _{g_{\mu _0,\mu _0}(t)}^{h_{\mu _0,\mu _0}(t)} K_2(h_{\mu _1,\mu _2}(t)-x)M_{\mu _0,\mu _0}(t,x)\textrm{d}x \\ \ge &  \mu _1 \int _{g_{\mu _0,\mu _0}(t)+\varepsilon _0}^{ h_{\mu _0,\mu _0}(t)-\varepsilon _0} K_1(h_{\mu _1,\mu _2}(t)-x)\textrm{d}x \inf _{x\in [g_{\mu _0,\mu _0}(t)+\varepsilon _0, h_{\mu _0,\mu _0}(t)-\varepsilon _0] } B_{\mu _0,\mu _0}(t,x) \\  &  +\mu _2 \int _{g_{\mu _0,\mu _0}(t)+\varepsilon _0}^{ h_{\mu _0,\mu _0}(t)-\varepsilon _0} K_2(h_{\mu _1,\mu _2}(t)-x)\textrm{d}x \inf _{x\in [g_{\mu _0,\mu _0}(t)+\varepsilon _0, h_{\mu _0,\mu _0}(t)-\varepsilon _0] } M_{\mu _0,\mu _0}(t,x) \\ = &  \mu _1 \int _{h_{\mu _1,\mu _2}(t)- h_{\mu _0,\mu _0}(t)+\varepsilon _0}^ {h_{\mu _1,\mu _2}(t)-g_{\mu _0,\mu _0}(t)-\varepsilon _0} K_1(x)\textrm{d}x \inf _{x\in [g_{\mu _0,\mu _0}(t)+\varepsilon _0, h_{\mu _0,\mu _0}(t)-\varepsilon _0] } B_{\mu _0,\mu _0}(t,x) \\  &  + \mu _2 \int _{h_{\mu _1,\mu _2}(t)- h_{\mu _0,\mu _0}(t)+\varepsilon _0}^ {h_{\mu _1,\mu _2}(t)-g_{\mu _0,\mu _0}(t)-\varepsilon _0} K_2(x)\textrm{d}x \inf _{x\in [g_{\mu _0,\mu _0}(t)+\varepsilon _0, h_{\mu _0,\mu _0}(t)-\varepsilon _0] } M_{\mu _0,\mu _0}(t,x) \\\ge &  \mu _1 \int _{H_{\infty }- h_{\mu _0,\mu _0}(t)+\varepsilon _0}^ {2h_0-\varepsilon _0} K_1(x)\textrm{d}x \inf _{x\in [g_{\mu _0,\mu _0}(t)+\varepsilon _0, h_{\mu _0,\mu _0}(t)-\varepsilon _0] } B_{\mu _0,\mu _0}(t,x) \\  &  + \mu _2 \int _{H_{\infty }- h_{\mu _0,\mu _0}(t)+\varepsilon _0}^ {2h_0-\varepsilon _0} K_2(x)\textrm{d}x \inf _{x\in [g_{\mu _0,\mu _0}(t)+\varepsilon _0, h_{\mu _0,\mu _0}(t)-\varepsilon _0] } M_{\mu _0,\mu _0}(t,x) \\\ge &  \mu _1 \int _{2\varepsilon _0}^ {2h_0-\varepsilon _0} K_1(x)\textrm{d}x \inf _{x\in [g_{\mu _0,\mu _0}(t)+\varepsilon _0, h_{\mu _0,\mu _0}(t)-\varepsilon _0] } B_{\mu _0,\mu _0}(t,x) \\ &  +\mu _2 \int _{2\varepsilon _0}^ {2h_0-\varepsilon _0} K_2(x)\textrm{d}x \inf _{x\in [g_{\mu _0,\mu _0}(t)+\varepsilon _0, h_{\mu _0,\mu _0}(t)-\varepsilon _0] } M_{\mu _0,\mu _0}(t,x) \\\ge &  \mu _1 \delta _0 \inf _{x\in [g_{\mu _0,\mu _0}(t)+\varepsilon _0, h_{\mu _0,\mu _0}(t)-\varepsilon _0] } B_{\mu _0,\mu _0}(t,x) \\  &  +\mu _2 \delta _0 \inf _{x\in [g_{\mu _0,\mu _0}(t)+\varepsilon _0, h_{\mu _0,\mu _0}(t)-\varepsilon _0] } M_{\mu _0,\mu _0}(t,x) \\:= &  \mu _1 \delta _0 \hat{m}_1(t)+ \mu _2 \delta _0 \hat{m}_2(t)\ge \delta _0\min \{\hat{m}_1(t), \hat{m}_2(t)\}(\mu _1+\mu _2 ), \end{aligned}$$
    which implies that
    $$\begin{aligned} \mu _1+\mu _2\le &  \frac{h_{\mu _1,\mu _2}(t_0+1)-h_{\mu _1,\mu _2}(t_0) }{ \delta _0 \min \{ \int _{t_0}^{ t_0+1}\hat{m}_1(t)\textrm{d}t,~~ \int _{t_0}^{ t_0+1}\hat{m}_2(t)\textrm{d}t \}} \\\le &  \frac{2L_* }{ \delta _0\min \{ \int _{t_0}^{ t_0+1}\hat{m}_1(t)\textrm{d}t,~~ \int _{t_0}^{ t_0+1}\hat{m}_2(t)\textrm{d}t \} }. \end{aligned}$$
    However, this leads to a contradiction due to the arbitrariness of $\mu _1+\mu _2$. Therefore, there exists $\overline{\mu } > 0$ such that spreading happens to (12) if $\mu _1+\mu _2 > \overline{\mu }$. Finally, we show that for fix $\mu _2$, there exists $\mu ^*$ such that vanishing happens for $0<\mu _1+ \mu _2\le \mu ^*$ and spreading happens for $\mu _1+ \mu _2>\mu ^*$. For any fixed $\mu _2>0$, we define
    $$ \mu ^*:=\sup \Sigma =\sup \{\mu ^0>0: \text{ vanishing } \text{ happens } \text{ for } \mu _1+\mu _2\in (0, \mu ^0]\}. $$
    Then, by Remark 4.7 and the above claim, we see that $0< \mu ^* < +\infty$. According to Corollary 3.6, $(B_{\mu _1,\mu _2}, M_{\mu _1,\mu _2}, g_{\mu _1,\mu _2}, h_{\mu _1,\mu _2})$ are increasing in $\mu _1>0$. This immediately gives that if $\mu _1 \in \Sigma$, then $\mu \in \Sigma$ for any $\mu <\mu _1$ and if $\mu _1 \not \in \Sigma$, then $\mu \not \in \Sigma$ for any $\mu > \mu _1$. Hence it follows that
    $$\begin{aligned} (0, \mu ^*) \subseteq \Sigma ,\ \ ( \mu ^*, +\infty ) \cap \Sigma =\emptyset . \end{aligned}$$
    To complete the proof, it remains to show that $\mu ^* \in \Sigma$. Suppose that $\mu ^* \not \in \Sigma$. Then it follows from Theorem 1.2 that $h_{\mu ^*-\mu _2,\mu _2}^{\infty }= -g_{\mu ^*-\mu _2,\mu _2}^{\infty } =+\infty$. Thus there exists $T>0$ such that $h_{\mu ^*-\mu _2,\mu _2 }(t) -g_{\mu ^*-\mu _2,\mu _2}(t)> 2L_*$ for $t\ge T$. Hence there exists $\epsilon >0$ such that $h_{\mu _1,\mu _2}(t) -g_{\mu _1,\mu _2}(t)> 2L_*$ for $0< \mu ^*-\mu _2-\mu _1 <\epsilon$. Therefore,
    $$\begin{aligned} h_{\mu _1 ,\mu _2}^\infty -g_{\mu _1 ,\mu _2}^\infty \ge h_{\mu _1 ,\mu _2}(t) -g_{\mu _1 ,\mu _2}(t)> 2L_*. \end{aligned}$$
    However, it follows from $\mu _1+\mu _2 < \mu ^*$ and the definition of $\mu ^*$ that $h_{\mu _1,\mu _2}^\infty -g_{\mu _1,\mu _2}^\infty <\infty$. This, combined with $\mathcal R_0>1$ and Lemma 4.1, implies that $h_{\mu _1,\mu _2}^\infty -g_{\mu _1,\mu _2}^\infty \le 2L_*$, which is a contradiction. Thus, $\mu ^* \in \Sigma$.
  - Similarly, for given $\mu _1$, we can also prove the existence of $\mu _*$ such that vanishing happens for $0<\mu _1+ \mu _2\le \mu _*$ and spreading happens for $\mu _1+ \mu _2>\mu _*$.

$$\square $$

## Numerical simulation and discussion

This section presents numerical simulations to verify the reliability of our theoretical results and to provide quantitative insights. To the best of our knowledge, there has been few numerical simulation studies on the classical nonlocal free boundary condition (as shown in the fourth and fifth lines of (7)). This paper appears to be the first to conduct numerical simulations on the more general free boundary condition (as shown in the fourth and fifth lines of (12)). Since the classical nonlocal free boundary condition is a special case of the more general condition, we hope that the numerical schemes presented here will inspire further research. The python codes for simulations are publicly available at https://github.com/ylou-polyu/main.git.

**Table 1:** Range of model parameters.

Parameters	Description	Range
$d_1$	the diffusion rate of birds	(0.1, 100)
$d_2$	the diffusion rate of mosquitoes	(0.01, 0.1)
$b_1$	the sum of the infectivity-to-susceptibility reversion rate and the natural mortality rate in birds	(0.1, 0.5)
$b_2$	the mosquito death rate	(0.016, 0.07)
$e_1$	the total number of birds	$(10^2, 10^3)$
$e_2$	the total number of mosquitoes	$(10^4, 10^6)$
$\alpha _M$	the WNv transmission probability per bite to mosquitoes	(0.5, 1.00)
$\alpha _B$	the WNv transmission probability per bite to birds	(0.02, 0.24)
$\beta _R$	the biting rate of mosquitoes on birds	(0.03, 0.16)
$\mu _1$	parameter of free boundary movement speed in birds	(0.001, 1)
$\mu _2$	parameter of free boundary movement speed in mosquitoes	(0.001, 1)

Based on the existing literature (Wonham et al. 2004; Moschini et al. 2017; Feng et al. 2022; Thomas and Urena 2001; Maidana and Yang 2009), we summarize ranges for parameters in Table 1. For simplicity, we choose $g_0=-h_0$. As shown in (2) and (3), we have

$$\begin{aligned} a_1=\frac{\alpha _B\beta _R}{e_1}, ~~~~ a_2=\frac{\alpha _M\beta _R}{e_1}, ~ ~~\mathcal R_0=\sqrt{\frac{a_1a_2e_1e_2}{b_1b_2}}. \end{aligned}$$

Let

$$\begin{aligned} R_*=\frac{a_1a_2e_1e_2}{(b_1+d_1)(b_2+d_2)}. \end{aligned}$$

For illustrative purpose, we choose the dispersal kernel functions $J_1$, $J_2$ and the boundary kernel functions $K_1$, $K_2$ to be:Clearly, $J_i$ satisfy the condition $(\textbf{J})$, $K_i$ satisfy the condition $(\textbf{K})$ and $K_i\in L^\infty ([0,\infty ))$, which implies $\limsup \limits _{y\rightarrow \infty }\frac{\int _0^y K_i(x)\textrm{d}x}{y}<\infty ~(i=1,2)$.

**Table 2:** Parameter values for different scenarios (A), (B) and (C).

Parameter	(A)	(B)	(C)
$\alpha _M$	0.5005	0.88	0.55
$\alpha _B$	0.091	0.16	0.1
$\beta _R$	0.15824	0.09	0.036
$a_1$	$1.44\times 10^{-5}$	$1.44\times 10^{-5}$	$1.44\times 10^{-5}$
$a_2$	$7.92\times 10^{-5}$	$7.92\times 10^{-5}$	$7.92\times 10^{-5}$
$b_1$	0.2	0.2	0.2
$b_2$	0.029	0.029	0.029
$d_1$	1	1	1
$d_2$	0.02	0.02	0.02
$e_1$	$10^2$	$10^3$	$0.25\times 10^3$
$e_2$	$10^4$	$10^5$	$0.25\times 10^5$
$\mu _1$	0.2	0.2	0.01
$\mu _2$	0.1	0.1	0.005
$h_0$	5	5	1
$\mathcal R_0$	$<1$	$>1$	$>1$
$R_*$	–	$>1$	$<1$
Longtime dynamics	Vanishing	Spreading	Vanishing

We first numerically validate the vanishing and spreading dynamics under various scenarios for Theorem 1.3, using the specific parameter sets (A), (B), (C) in Table 2.

### Example 5.1

**Fig. 1:**
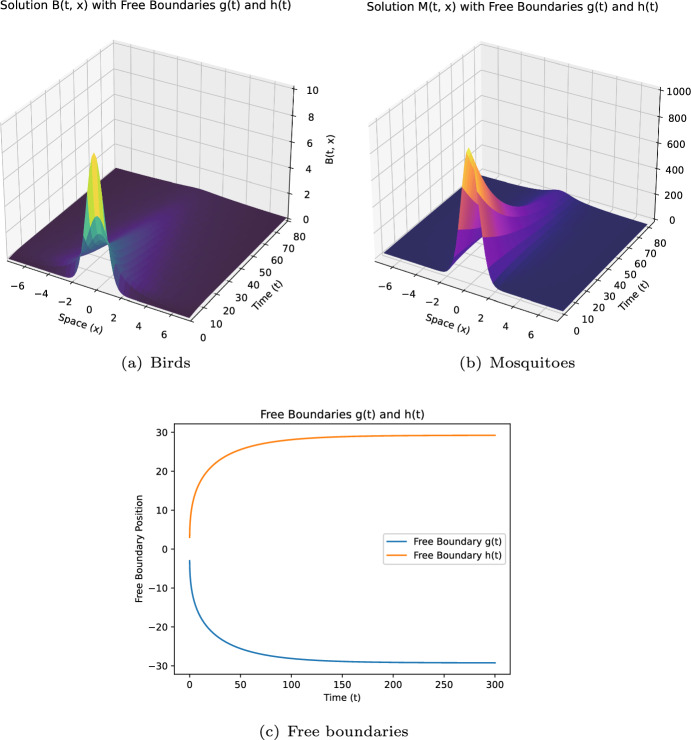
Solution evolution for scenario (A)

*(Vanishing dynamics for Theorem* 1.3*-(i))* Based on the parameter set (A) in Table 2, we find $\mathcal {R}_0 < 1$. According to Theorem 1.3-(i), vanishing phenomenon occurs for (12). Numerical simulations confirm this theoretical prediction, with results for the free boundaries shown in Fig. 1c, and solutions for bird and mosquito population densities depicted in Figs. 1a and 1b.

### Example 5.2

**Fig. 2:**
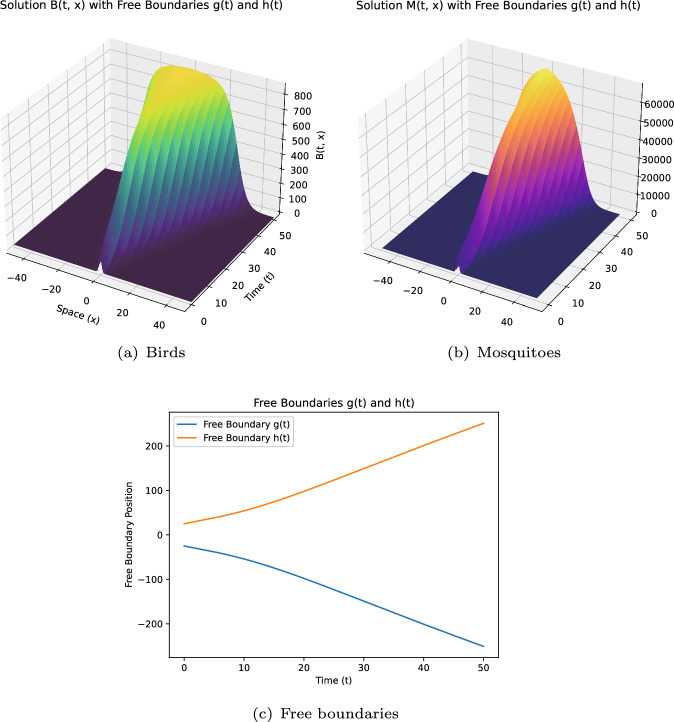
Solution evolution for scenario (B).

*(Spreading dynamics for Theorem* 1.3*-(iii))* When we choose the parameter set (B) in Table 2, we have $\mathcal {R}_0 > 1$ and $R_* > 1$. According to Theorem 1.3-(iii), spreading phenomenon will occur for (12). This is confirmed by numerical simulations in Fig. 2.

### Example 5.3

**Fig. 3:**
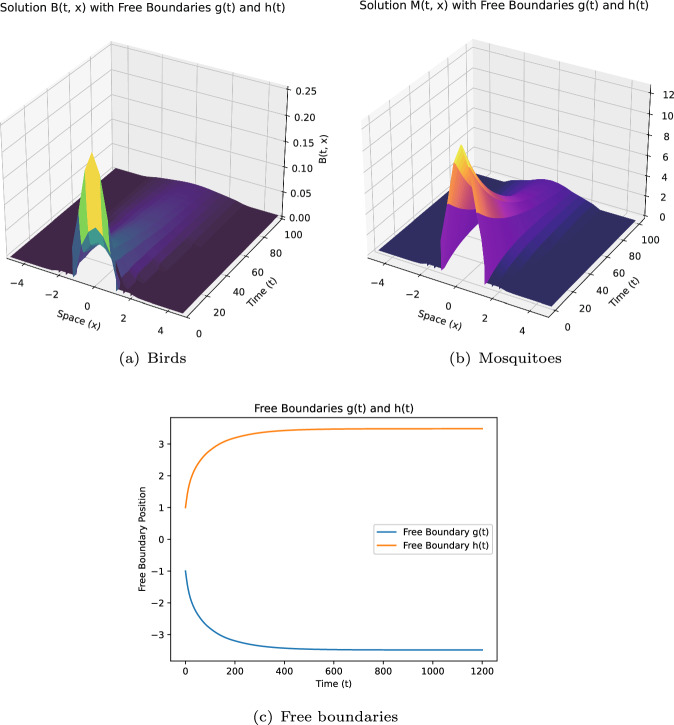
Solution evolution for scenario (C)

*(Vanishing dynamics for Theorem* 1.3*-(iv))* Using the specific values of the parameter set (C) in Table 2, it can be seen that $\mathcal {R}_0 > 1$, $R_* < 1$, and both $\mu _1 + \mu _2$ and the initial value of $h_0$ are sufficiently small. These conditions, combined with Theorem 1.3-(iv), show that the vanishing phenomenon will occur for (12). Numerical simulations fully support this theoretical inference, as shown in Figs. 3.

Next, we evaluate the impact of the initial boundary value $h_0$ and the efficacy of mosquitoes in pushing the boundary, represented by $\mu _2$, on the dynamics of (12). Using baseline parameter values from set (C) as follows:

$$ a_1 = 1.44 \times 10^{-5}, \quad a_2 = 7.92 \times 10^{-5}, \quad b_1 = 0.2, \quad b_2 = 0.029, $$

$$ d_1 = 1, \quad d_2 = 0.02, \quad e_1 = 0.25 \times 10^{3}, \quad e_2 = 0.25 \times 10^{5}, $$

we vary $h_0$ and $\mu _2$ as outlined in Table 3 to observe changes in the dynamic behavior of the numerical simulations. We also examine whether these results align with our theoretical findings in Theorem 1.3.

**Table 3:** Parameter values of $h_0$, $\mu _1$, $\mu _2$, with varying $h_0$ and $\mu _2$ values.

$h_0$	$\mu _1$	$\mu _2$	Longtime dynamics
2	0.01	0.005	Vanishing
10			Spreading
20			Spreading
1	0.01	0.005	Vanishing
		0.025	Spreading
		0.05	Spreading

**Fig. 4:**
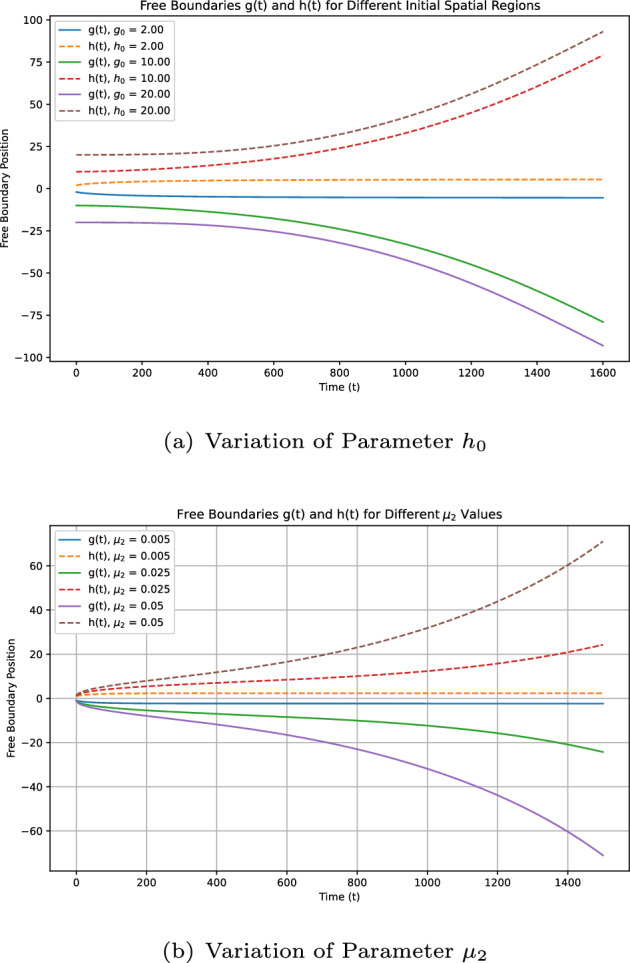
Effect of $h_0$ and $\mu _2$ on the evolution of free boundaries

### Example 5.4

*(Effect of initial boundary* $h_0$*)* From the given parameter values, we verify that $\mathcal {R}_0 > 1$ and $R_* < 1$, corresponding to Theorem 1.3-(iv). As shown in the second, third, and fourth rows of Table 3, we set the initial value $h_0$ to 2, 10, and 20, respectively. The numerical simulations reveal dynamic behaviors of vanishing, spreading, and spreading phenomena (see Fig. 4a), strongly supporting the conclusion of Theorem 1.3-(iv)-(a). Specifically, for fixed $\mu _1$ and $\mu _2$, vanishing occurs when the initial value $h_0$ is sufficiently small.

This phenomenon aligns with the spatial constraint effect in population ecology: when the initial habitat area is extremely limited, the population’s resource acquisition capacity is insufficient to meet basic survival requirements, posing significant challenges to species persistence.

### Example 5.5

*(Effect of* $\mu _2$*)* For given $\mu _1 = 0.01$, as shown in the fifth, sixth, and seventh rows of Table 3, we set $\mu _2$ to 0.005, 0.025, and 0.05, respectively. The numerical simulations demonstrate vanishing, spreading, and spreading behaviors (see Fig. 4b). This supports Theorem 1.3-(iv)-(c), which states that for a fixed $\mu _1$, vanishing occurs when $\mu _2$ is small, while spreading occurs as $\mu _2$ increases. Additionally, this highlights the significant impact of the infected mosquito term and its parameter $\mu _2$ on the free boundary conditions, which fully supports the plausibility of our modeling.

Although the global dynamics of the model system have been thoroughly established, the current model formulation remains highly simplified. A more realistic WNv model would incorporate additional important factors, such as seasonality and time delays, both of which are critical for determining transmission dynamics. Seasonal variations in temperature, precipitation, and other environmental conditions strongly influence the population dynamics of mosquito vectors and avian hosts, thereby affecting WNv transmission. Incorporating seasonality, typically through time-dependent and periodic parameters for mosquito birth, mortality, or biting rates, can substantially enhance the biological realism of the model. Time delays, resulting from biological processes such as the incubation period within mosquitoes, mosquito maturation, and the latent period in birds, are also important in the various processes of the transmission cycle. Recent studies on free-boundary problems with periodic parameters (Du et al. 2013), time delays (Sun and Fang 2019), and related research provide valuable frameworks for addressing these complexities. Extending our model to include seasonality and time-delay effects represents a promising direction for future investigation.

## References

[CR1] Abdelrazec A, Lenhart S, Zhu HP (2014) Transmission dynamics of West Nile virus in mosquitoes and corvids and non-corvids. J Math Biol 68:1553–1582 23652768 10.1007/s00285-013-0677-3

[CR2] Bao XX, Li WT, Shen WX (2016) Traveling wave solutions of Lotka-Volterra competition systems with nonlocal dispersal in periodic habitats. J Differ Equ 260:8590–8637

[CR3] Bao XX, Shen W (2017) Criteria for the existence of principal eigenvalues of time periodic cooperative linear systems with nonlocal dispersal. Proc Amer Math Soc 145:2881–2894

[CR4] Berestycki H, Coville J, Vo H (2016) Persistence criteria for populations with non-local dispersion. J Math Biol 72:1693–1745 26162491 10.1007/s00285-015-0911-2

[CR5] Ben-Nathan D, Porgador A, Yavelsky V et al (2006) Models of West Nile virus disease. Drug Discov Today Dis Model 3:49–54

[CR6] Berestycki H, Coville J, Vo H (2016) On the definition and the properties of the principal eigenvalue of some nonlocal operators. J Funct Anal 271:2701–2751

[CR7] Bowman C, Gumel AB, Wu J et al (2005) A mathematical model for assessing control strategies against West Nile virus. Bull Math Biol 67:1107–1133 15998497 10.1016/j.bulm.2005.01.002

[CR8] Caffarelli L, Salsa S (2005) A Geometric Approach to Free Boundary Problems, Grad. Stud. Math. 68, American Mathematical Society, Providence, RI

[CR9] Cao J, Du Y, Li F, Zhou ML (2019) The dynamics of a Fisher-KPP nonlocal diffusion model with free boundaries. J Funct Anal 277:2772–1814

[CR10] Ceausu E, Erscoiu S, Calistru P et al (1997) Clinical manifestations in the West Nile virus outbreak. Rom Arch Microbiol Immunol 48:3–11 9836323

[CR11] Clobert J, Baguette M, Benton T et al (2012) Dispersal Ecology and Evolution. Oxford University Press, Oxford, UK

[CR12] Du Y, Guo Z, Peng R (2013) A diffusive logistic model with a free boundary in time-periodic environment. J Func Anal 265:2089–2142

[CR13] Du Y, Li F, Zhou ML (2021) Semi-wave and spreading speed of the nonlocal Fisher-KPP equation with free boundaries. J Math Pures Appl 154:30–66

[CR14] Du Y, Lin Z (2010) Spreading-Vanishing dichotomy in the diffusive logistic model with a free boundary. SIAM J Math Anal 42:377–405

[CR15] Du Y, Long X, Ni W, Quirós F (2024) Precise rate of propagation for the nonlocal Fisher-KPP model with a weight in the free boundary condition, preprint

[CR16] Du Y, Ni W (2020) Analysis of a West Nile virus model with nonlocal diffusion and free boundaries. Nonlinearity 33:4407–4448

[CR17] Du Y, Ni W (2022) Spreading speed for some cooperative systems with nonlocal diffusion and free boundaries, part 1: Semi-wave and a threshold condition. J Differ Equ 308:369–420

[CR18] Du Y, Wang M, Zhao M (2022) Two species nonlocal diffusion systems with free boundaries, Disc Con Dynam Syst-A, **42** , 1127-1162

[CR19] Feng CX, Lewis MA, Wang CC et al (2022) A Fisher-KPP model with a nonlocal weighted free boundary: analysis of how habitat boundaries expand, balance or shrink. Bull Math Biol 84(3):34 35084578 10.1007/s11538-022-00995-8

[CR20] Garnier J (2011) Accelerating solutions in integro-differential equations. SIAM J Math Anal 43:1955–1974

[CR21] Hutson V, Martinez S, Mischaikow K et al (2003) The evolution of dispersal. J Math Biol 47:483–517 14618377 10.1007/s00285-003-0210-1

[CR22] Lewis MA, Renclawowicz J, van den Driessche P (2006) Traveling waves and spread rates for a West Nile virus model. Bull Math Biol 68:3–23 16794919 10.1007/s11538-005-9018-z

[CR23] Li WT, Sun YJ, Wang ZC (2010) Entire solutions in the Fisher-KPP equation with nonlocal dispersal. Nonlinear Anal Real World Appl 11(4):2302–2313

[CR24] Lin ZG, Zhu HP (2017) Spatial spreading model and dynamics of West Nile virus in birds and mosquitoes with free boundary. J Math Biol 75:1381–1409 28378145 10.1007/s00285-017-1124-7

[CR25] Long X, Du YH, Ni WJ, Yi TS (2024) Dynamics Of the Nonlocal KPP Equation: Effects of a New Free Boundary Condition. J Differ Equ 413:557–605

[CR26] Moschini P, Bisanzio D, Pugliese A (2017) A seasonal model for West Nile virus. Math Model Nat Phenom 12(2):58–83

[CR27] Nagy M, Akos Z, Biro D et al (2010) Hierarchical group dynamics in pigeon flocks. Nature 464:890–893 20376149 10.1038/nature08891

[CR28] Nathan R, Klein E, Robledo-Arnuncio JJ et al (2012) Dispersal kernels: Review. In: Clobert J, Baguette M, Benton TG, Bullock JM (eds) Dispersal Ecology and Evolution. Oxford University Press, Oxford, UK, pp 187–210

[CR29] Sun N, Fang J (2019) Propagation dynamics of Fisher-KPP equation with time delay and free boundaries. Calc Var 58:148

[CR30] Thomas DM, Urena B (2001) A model describing the evolution of West Nile-like encephalitis in New York City. Math Comput Model 34(7–8):771–781

[CR31] Tsai TF, Popovici F, Cernescu C et al (1998) West Nile encephalitis epidemic in Southeastern Romania. Lancet 352:761–771 10.1016/s0140-6736(98)03538-7 9737281

[CR32] Wang BG, Qiang L, Wang ZC (2020) An almost periodic Ross-Macdonald model with structured vector population in a patchy environment. J Math Biol 80:835–863 31655877 10.1007/s00285-019-01443-3

[CR33] Wang ZG, Nie H, Du YH (2019) Spreading speed for a West Nile virus model with free boundary. J Math Biol 79:433–466 31016334 10.1007/s00285-019-01363-2

[CR34] Wonham MJ, De-Camino-Beck T, Lewis MA (2004) An epidemiological model for West Nile virus: invasion analysis and control applications. Proc R Soc Lond B 271:501–507 10.1098/rspb.2003.2608 PMC1691622 15129960

[CR35] http://www.cdc.gov/ncidod/dvbid/westnile/survcontrol.htm

[CR36] Maidana N, Yang H (2009) Spatial Spreading of West Nile Virus Described by Traveling Waves. J Theor Biol 258:403–417 19167405 10.1016/j.jtbi.2008.12.032

